# *Vaccinium myrtillus* L. Leaf Waste as a Source of Biologically Potent Compounds: Optimization of Polyphenol Extractions, Chemical Profile, and Biological Properties of the Extracts

**DOI:** 10.3390/pharmaceutics16060740

**Published:** 2024-05-30

**Authors:** Muna Rajab Elferjane, Violeta Milutinović, Milica Jovanović Krivokuća, Mohammad J. Taherzadeh, Witold Pietrzak, Aleksandar Marinković, Aleksandra A. Jovanović

**Affiliations:** 1 Faculty of Nursing and Health Sciences, University of Misurata, Alshowahda Park, 3rd Ring Road, Misurata 2478, Libya; 20204009@estudent.tmf.bg.ac.rs; 2 Faculty of Technology and Metallurgy, University of Belgrade, Karnegijeva 4, 11000 Belgrade, Serbia; marinko@tmf.bg.ac.rs; 3 Faculty of Pharmacy, University of Belgrade, Vojvode Stepe 450, 11000 Belgrade, Serbia; violeta.milutinovic@pharmacy.bg.ac.rs; 4 Institute for the Application of Nuclear Energy INEP, University of Belgrade, Banatska 31b, 11080 Belgrade, Serbia; milicaj@inep.co.rs; 5 Swedish Centre for Resource Recovery, University of Borås, Allégatan 61, 503 37 Borås, Sweden; mohammad.taherzadeh@hb.se; 6 Department of Fermentation and Cereals Technology, Wrocław University of Environmental and Life Sciences, Norwida 25, 50-375 Wrocław, Poland; witold.pietrzak@upwr.edu.pl

**Keywords:** anti-inflammatory activity, antioxidant activity, cytotoxicity, optimization, polyphenolic compounds, bilberry, waste

## Abstract

The aims of the present research include (1) optimization of extraction from *Vaccinium myrtillus* leaf waste via investigation of plant material:medium ratio, extraction medium, and extraction period, employing extractions at room and high temperatures, or using ultrasound and microwaves (M, HAE, UAE, and MAE, respectively), (2) physicochemical characterization, and (3) investigation of extract biological potential. The statistical analysis revealed that optimal levels of parameters for the greatest polyphenolic yield were a proportion of 1:30 g/mL, ethyl alcohol 50% (*v*/*v*) during 2 min of microwave irradiation. By LC-MS analysis, 29 phenolic components were detected; HAE showed the highest richness of almost all determined polyphenols, while chlorogenic acid and quercetin 3-*O*-glucuronide were dominant. All extracts showed a high inhibition of *Staphylococcus aureus* growth. The effect of different parameters on extracts’ antioxidant capacity depended on the used tests. The extracts also showed a stimulative influence on keratinocyte viability and anti-inflammatory activity (proven in cell-based ELISA and erythrocyte stabilization assays). The extraction procedure significantly affected the extraction yield (MAE ≥ maceration ≥ UAE ≥ HAE), whereas conductivity, density, surface tension, and viscosity varied in a narrow range. The presented research provides evidence on the optimal extraction conditions and technique, chemical composition, and antioxidant, antimicrobial, anti-inflammatory, and keratinocyte viability properties of bilberry extracts for potential applications in pharmacy and cosmetics.

## 1. Introduction

*Vaccinium myrtillus* L. (bilberry) is a shrub from the Ericaceae family and genus *Vaccinium* and its habitat is mountainous regions in Europe, Asia, and North America. Its fruit and leaves have significance in various industries as the ingredients in foods, dietetic supplements, and pharmacological and cosmetic formulations [1]. *V. myrtillus* leaves contain valuable compounds, including phenolic acids, flavonoids, procyanidins, anthocyanins, stilbene derivatives, tannins, iridoid compounds, fatty acids, triterpenes, fibrous components, micronutrients, and macronutrients [1,2,3,4]. In folk medicine, bilberry extracts prepared using leaves were employed in the treatment of disorders in the urinary system because of the presence of compounds with antimicrobial and/or astringent activities [1]. Additionally, the extracts are able to ameliorate the symptoms of diabetes [5,6], contributing to the decrease of free radicals, inflammation, and dyslipidemia. Namely, the aforementioned roles in relieving the symptoms of diabetes are possible due to the extract’s antioxidant, anti-inflammatory, and lipid-lowering properties [1,7,8]. According to the literature, the leaf extract can be a major skin-rejuvenating supplement due to its ability to enhance the expression of genes in fibroblast cells (such as glutamate decarboxylase) and to increase cell proliferation and synthesis of different compounds, including glutathione and hyaluronic acid [9]. The mentioned extract also causes the inhibition of the various enzymes that have an important role in the formation of wrinkles, including collagenase and elastase, the decrease in the synthesis of melanin, and the suppression of the release of histamine [7,9].

There are plenty of extraction procedures that can extract biologically active components from plant matrix, however, they differ in terms of the extraction speed and efficiency, yield of target molecules, and consumption of solvent and energy [10]. Maceration represents a conventional technique that is suitable for thermosensitive components using various extraction solvents and simple and inexpensive devices. Nevertheless, maceration possesses several disadvantages, including poor extraction efficiency, a long period, as well as a high amount of herbal matrix and medium, consequently resulting in a negative influence on the environment [10,11]. Hence, the application of modern methods for extraction, including subcritical and supercritical fluid extractions, and heat-, enzyme-, ultrasound-, or microwave-assisted extractions have been evaluated in recent times. The mentioned novel procedures provide various advantages, such as reduced extraction medium amount, a shortened period, increased extraction efficiency, and reduced negative environmental effects, i.e., sustaining the concept of green extraction [11]. Additionally, the isolation of different bioactive and functional compounds can be a good way to make use of herbal waste or by-products [12]. Specifically, carotenoids have been successfully extracted using waste of vegetables and fruits, including grape, pomegranate, and pumpkin seeds, banana, grape, and tomato peels, or apricot bagasse and pomace [13], polyphenols from black chokeberry, grape, olive, and artichoke waste, pine sawdust, or aromatic plant dust [14,15,16,17], pectin from watermelon rind or passion fruit peel [18,19], enzymes from potato, banana, pineapple, lemon, or mango peels [20], etc. Mosoarca et al. [21] have shown that the bioadsorbent material based on *V. myrtillus* leaf powder (as a waste) is very efficacious for removing methylene blue dye from water. Namely, regarding regulations on the quality of tea, herbal tea, and their products of the Republic of Serbia, plant material with a particle size lower than 0.5 mm (herbal dust) cannot be a constituent part of the tea formulations for the market, trade, and sale [22].

Based on the above-stated background and the past use of bilberry leaves in traditional therapy, the research goals were the optimization of the extraction of *V. myrtillus* waste (dust) from leaves by the examination of the following parameters: plant material:medium ratio, extraction medium, and extraction period by employing four methods, including extractions at room temperature (maceration) and high temperature (heat-assisted extraction, HAE) and using ultrasound waves of an ultrasound probe (ultrasound-assisted extraction, UAE) and microwaves in a microwave reactor (microwave-assisted extraction, MAE). The selection of an appropriate extraction procedure is important from the aspect of the industrial requirements (increased extraction efficiency and reduced time and costs). On the other hand, the quality of the final extracts (the highest content of target compounds without the presence of ballast components, allergens, heavy metals, impurities, etc.) is of essential importance for future application. Namely, bilberry leaves represent a big part of waste biomass, so their processing would contribute to more complete waste management and biodiversity preservation. The optimal extraction conditions for bilberry leaf by-product were determined via polyphenol content using an experimental design. The optimized LC-MS method was used for the qualitative and quantitative analyses of individual polyphenols in four selected extracts. The analysis of the amounts of total flavonoid compounds, condensed tannin compounds, and proteins, Fourier transform infrared (FT-IR) spectra, measurement of physical properties, radical-scavenging and ion reduction potential, and antibacterial, antifungal, anti-inflammatory, and skin cell viability properties of the extracts were examined as well. Thus, the research gives an insight into the optimal extraction conditions and method (for achieving the highest polyphenol yield), physicochemical characteristics, and biological potential of the extracts from *V. myrtillus* waste that can be integrated into pharmacological, dermo-cosmetic, or cosmetic preparations for skin disorders.

## 2. Materials and Methods

### 2.1. Herbal Material, Reagents, and Standards

The *V. myrtillus* by-product from leaves was plant dust. Namely, the size of the plant material was 0.3 mm, as a result of the comminution of the starting herbal matrix. The obtained powder is waste (by-product or dust), thus, regarding regulations on the quality of tea, herbal tea, and their products of the Republic of Serbia, it cannot be a constituent part of the tea formulations for the market, trade, and sale [22].

Ultrapure water was obtained using the Simplicity UV^®^ water purification system (Merck Millipore, Merck KGaA, Darmstadt, Germany), whereas ultrapure water employed in the LC-MS method was obtained in the TKA water purification system (Thermo Fisher Scientific, Niederelbert, Germany). Analytical standards (caffeic acid, chlorogenic acid, quercetin 3-*O*-rutinoside, quercetin 3-*O*-glucoside, gallic acid, procyanidin C1, and procyanidin B2), as well as Coomassie^®^ brilliant blue G-250, Folin–Ciocalteu reagent, and phosphoric acid, were obtained from Sigma-Aldrich (Merck Group, Merck KGaA, Darmstadt, Germany), quercetin 3-*O*-galactoside from Carl Roth (Karlsruhe, Germany), while quercetin 3-*O*-glucuronide and quercetin 3-*O*-rhamnoside from HWI Analytik (Ruelzheim, Germany) and *p*-coumaric acid from Fluka (Buchs, Switzerland). Ethanol (p.a.), sodium carbonate, sulfuric acid, and sodium chloride were from Fisher Scientific (Loughborough, Leicestershire, United Kingdom), sodium nitrite was from Alkaloid (Skopje, Macedonia), thiazolyl blue tetrazolium bromide (MTT), aluminum chloride, phosphate-buffered saline (PBS), 6-hydroxy-2,5,7,8-tetramethylchroman-2-carboxylic acid (Trolox), 2,2′-azino-bis(3-ethylbenzothiazoline-6-sulfonic acid) (ABTS), 2,2-diphenyl-1-picrylhydrazyl (DPPH), 2,4,6-Tris(2-pyridyl)-s-triazine (TPTZ), sodium acetate, sodium dodecyl sulfate (SDS), bovine serum albumin (BSA), tetramethylbenzidine (TMB), sodium oxalate, and acetic acid were from Sigma-Aldrich (Darmstadt, Germany), iron(III) chloride, potassium ferricyanide, methanol, acetone, bacterial lipopolysaccharide (LPS; *Escherichia coli* 055:B5), catechin, iron(II) sulfate, anti-goat IgG secondary antibodies, and solvents acetonitrile and formic acid (LC-MS grade) were from Sigma-Aldrich (Saint Louis, MO, USA), neocuproin was from Acros Organics (Geel, Antwerpen, Belgium), vanillin and hydrochloric acid were from Merck (Darmstadt, Germany), cuprum chloride was from Fluka (Seelze, Germany), ammonium acetate was from Zorka Pharma (Šabac, Serbia), diclofenac ampoule 75 mg/3 mL was from Galenika (Belgrade, Serbia), 1M sodium hydroxide was from Alfapanon (Bački Petrovac, Serbia), potassium persulfate was from Centrohem (Stara Pazova, Serbia), and mediums in Petri dishes—Sabouraud dextrose agar plate and Mueller–Hinton agar plate—were purchased from Promedia (Kikinda, Serbia). Dulbecco’s modified Eagle’s medium and Ham’s F-12 nutrient mixture (1:1 mixture, DMEM/F12 cell culture medium) were from Pan-Biotech (Aidenbach, Germany). Goat anti-human IL-6 was from R&D Systems (Minneapolis, MN, USA), while biotinylated anti-goat IgG secondary antibodies and avidin-biotinylated peroxidase complex (ABC) were from Vector Laboratories (Burlingame, CA, USA). Immortalized human keratinocytes (HaCaT cell line) were from the Institute for Biological Research “Siniša Stanković”, National Institute of the Republic of Serbia, University of Belgrade, Belgrade, Serbia. RPMI 1640 medium was from GIBCO BRL (Thermo Fisher Scientific, Waltham, MA, USA), whereas 10% fetal bovine serum (FBS) and 1% mixture of antibiotic and antimycotic were purchased from Capricorn Scientific (Ebsdorfergrund, Germany).

### 2.2. Extraction Procedures

#### 2.2.1. Maceration

Maceration was performed at 25 °C using the incubator shaker KS 4000i control (IKA, Staufen, Germany) at three plant material:medium ratios (1:10, 1:20, and 1:30 g/mL), in three different extraction mediums (water and 50 and 70% ethanol), during different extraction periods (30, 45, and 60 min). The samples were obtained according to plant material:medium ratios (1 g of herbal matrix with 10, 20, and 30 mL of the extraction medium). The Erlenmeyer flask was covered using aluminum foil with the aim of preventing light exposure degradation and vaporization of the extraction medium.

#### 2.2.2. Heat-Assisted Extraction (HAE)

HAE was carried out by employing the same parameters as presented in the previously mentioned extraction procedure (Section 2.2.1) for 15, 30, and 45 min. The temperature in the incubator shaker was 80 °C.

#### 2.2.3. Ultrasound-Assisted Extraction (UAE)

UAE was carried out by employing the ultrasound probe (750 W, 20 kHz, and 13 mm, Sonopuls, Bandelin, Berlin, Germany). The value of amplitude was 60%, while the temperature was maintained at 25–27 °C due to the ice coating of the bottle with the extract during sonication. The previous experiment showed that the highest content of polyphenols was reached using 60% amplitude. The same parameters were employed as in the above-described extraction techniques for 5, 15, and 30 min.

#### 2.2.4. Microwave-Assisted Extraction (MAE)

MAE was performed in a Monowave 300 microwave reactor (Anton Paar GmbH, Graz, Austria). The temperature was 100 °C. The previous experiment showed that the highest concentration of polyphenols was reached at 100 °C. Herbal matrix (1, 0.5, or 0.33 g) and 10 mL of the extraction medium in a closed reactor vial (lower volume was used because of the technical characteristics of the microwave reactor) were mixed and stirred at 600 rpm. The same parameters were employed as in the above-described extraction techniques for 1, 2, and 3 min.

The samples were filtered using a cellulose filter paper with a pore size of 0.45 µm and kept in the refrigerator (4 °C) until future experiments.

#### 2.2.5. Freeze-Drying of the Samples

The selected liquid *V. myrtillus* leaf waste extracts (prepared using the extraction conditions to reach the highest polyphenolic yield in all used procedures) were lyophilized to prepare dried extracts for FT-IR analysis and investigation of antibacterial, antifungal, and anti-inflammatory properties, as well as influence on keratinocyte viability. Organic solvent, i.e., ethanol, was eliminated in a vacuum rotary evaporator (Heizbad Hei-VAP, Heidolph, Heidelberg, Germany). The temperature was 50 °C, while the pressure was 50 mbar. The extracts were lyophilized using Alpha 2-4 LSCplus (Christ, Osterode am Harz, Germany). The temperature was −75 °C, while the pressure was 0.011 mbar for 24 h. In an additional hour, the temperature was −65 °C, whereas the pressure was 0.054 mbar.

### 2.3. Chemical Characterization of the Extracts

#### 2.3.1. Total Polyphenols

The total polyphenol content (TPC) in the extracts was measured spectrophotometrically employing the Folin–Ciocalteu assay with a slight modification [23]. The liquid sample in a volume of 20 µL was mixed with water (1200 µL) and the Folin–Ciocalteu reagent previously diluted with water (100 µL). Subsequently, 20% sodium carbonate solution (300 µL) and 380 µL of water were added. The mixture was kept in the dark for 2 h and the absorbance was measured at a wavelength of 765 nm. The calibration curve was obtained using gallic acid. The polyphenol content was presented as mg gallic acid equivalents (GAE) per g of dried herbal material.

#### 2.3.2. Total Flavonoids

The total flavonoid content (TFC) in four *V. myrtillus* leaf extracts (obtained under the optimal conditions determined using the experimental design in all used procedures for the extraction) was measured using a colorimetric test [24]. The sample in a volume of 250 µL and 5% sodium nitrite solution (75 µL) were mixed with 1250 µL of water. Aluminum chloride solution (150 µL, 8.8%) was poured at the 5th minute and sodium hydroxide solution (500 µL, 1M) and water (up to 3 mL) were added at the 6th minute. Subsequently, the absorbance was read at a wavelength of 510 nm. The calibration curve was obtained using catechin monohydrate. The flavonoid content was presented as mg catechin equivalents (CE) per g of dried herbal material.

#### 2.3.3. Condensed Tannins

The concentration of condensed tannins in four bilberry leaf samples (the same extracts as in the case of the assay for determination of total flavonoids) was measured using an assay with vanillin and HCl [25]. The sample in a volume of 200 µL was mixed with vanillin in methanol (4%, 1500 µL) and 750 μL of concentrated HCl (37%). The mixture was kept in the dark for 15 min. The absorbance was read at a wavelength of 500 nm. The calibration curve was obtained using catechin monohydrate. The results were presented as mg CE per g of dried herbal material.

#### 2.3.4. Total Proteins

The concentration of total proteins of four bilberry leaf extracts (the same extracts as in the case of total flavonoids and condensed tannins) was measured in an assay described by Bradford [26]. The Bradford dye reagent consisted of 100 mg of Coomassie brilliant blue G-250, 50 mL of ethyl alcohol, and phosphoric acid (85%, 100 mL), made up to 1000 mL with water. To the Bradford dye solution (volume of 2500 µL) was added extract (50 µL). The absorbance was read at a wavelength of 595 nm. The calibration curve was obtained using albumin. The total protein content was presented as mg of proteins per g of dried herbal material.

#### 2.3.5. FT-IR Spectroscopy

The selected lyophilized *V. myrtillus* extracts were characterized in a Nicolet™ iS™ 10 FTIR Spectrometer (Thermo Fisher Scientific, Uppsala, Sweden). FT-IR spectra were obtained in the following range: 4000 and 400 cm^−1^.

#### 2.3.6. LC-MS Analysis

Four *V. myrtillus* leaf extracts, selected according to the highest TPC, previously determined by the spectrophotometric assay [23], were further analyzed by LC-MS. The extracts were obtained using all four extraction procedures (maceration, HAE, UAE, and MAE) under the optimal extraction conditions. For the LC-MS analysis, an Agilent LC/MS System 1260/6130 (Agilent Technologies, Waldbronn, Germany) with diode-array detector (DAD) and single quadrupole API-ESI MSD, equipped with ChemStation software Rev. B.04.03-SP1 and Zorbax SB-Aq column (150 × 3.0 mm; 3.5 µm particle size, Agilent Technologies), was used. Firstly, liquid extracts (1.5 mL) were slowly dried in an RVC 2-18 CDplus (Christ, Osterode am Harz, Germany) at 50 °C for 6 h. The obtained dried extracts were resolved in methanol (5 mg/mL) and filtered (through a cellulose membrane filter, 0.45 µm). The binary mobile phase (MF) included MF A (water solution of formic acid, 0.1%, *v*/*v*) and MF B (acetonitrile). The operating column temperature was 25 °C, the flow rate 0.35 mL/min, and the injection volume of 2 μL. DAD spectra were recorded at 280, 320, and 350 nm, while mass spectra (MS) in the negative ion mode (in the full-scan mode in the range of *m*/*z* 80–2500 and the selected ion monitoring (SIM) mode of *m*/*z* 463 and 477 for two flavonoids). The ion source parameters were optimized according to previous work [27].

Eight detected compounds were identified using commercially available standards (retention time (Rt), UV, and MS spectra comparison). The structures of the other detected compounds were characterized to the highest reliable level on the basis of their UV and/or MS spectra (literature data comparison). The external standard method was used for the quantitative analysis, and the contents of the detected compounds were calculated based on the peak areas obtained by DAD (at 280, 320, and 350 nm) or MSD (in SIM mode for two flavonoids) using calibration curves obtained for nine standards. Compounds were identified based on the comparison of their UV and MS (obtained in full-scan mode) spectra to those of commercially available standards, or the structure was characterized to the highest reliable level by analyzing their UV or MS spectra. The peak areas obtained by DAD (at 280, 320, and 350 nm for phenolic constituents) or MSD (in SIM mode for two flavonoids) were used for the quantitative analysis by the external standard method. Calibration curves were obtained using seven different concentrations of nine standard compounds. Regression equations, correlation coefficients (r^2^), linear ranges, and limits of detection (LODs) and quantification (LOQs) are given in Table 1. LODs and LOQs were calculated from signal-to-noise ratios of 3 and 10, according to the International Conference on Harmonization guidelines (ICH, 2005) [28]:

LOD = 3.3 × σ/a(1)

LOQ = 10 × σ/a(2)

### 2.4. Antioxidant Activity Tests

The antioxidant activity of all obtained *V. myrtillus* samples was measured using various antioxidant assays with dissimilar principles (radical-scavenging potential—ABTS and DPPH assays, and ion reduction potential—FRAP and CUPRAC assays).

#### 2.4.1. ABTS Method

The ABTS test was employed to investigate the extract capacity of the ABTS free radical neutralization [29]. The ABTS powder (19 mg) was dissolved in water (5 mL) and mixed with 88 µL of potassium persulfate solution. The mixture was kept at 4 °C for 24 h. The working mixture was diluted using ethanol to achieve ~0.700 at a wavelength of 734 nm. Diluted liquid extract (20 µL, in a 1:9 ratio using the extraction medium) was added to the ABTS working mixture (2 mL) and left in the dark for 6 min. The absorbance values were read at a wavelength of 734 nm. The radical-scavenging capacity (∆A) was determined by employing the following equation:

∆A = A_0_ − A_x_ (3)

A_0_—absorbance of the ABTS working mixture and the extraction solvent; A_x_—absorbance of the ABTS working mixture and bilberry extract. The calibration curve was obtained using Trolox. The results were shown as µmol Trolox equivalents (TE) per g of dried herbal material.

#### 2.4.2. DPPH Method

The DPPH test was also performed in the determination of the extract capacity of DPPH free radical neutralization [24]. The DPPH powder was dissolved in ethanol to achieve ~0.800 at a wavelength of 517 nm. Various concentrations of liquid extract (100 μL) were added to 2 mL of DPPH working mixture and left in the dark for 20 min. The absorbance values were determined at a wavelength of 517 nm. The level of DPPH radical inhibition was determined by employing the following equation:

% inhibition = (A_0_ − A_x_) × 100/A_0_ (4)

A_0_—absorbance of the DPPH working mixture and the extraction solvent, A_x_—absorbance of the DPPH working mixture and bilberry extract. The DPPH-radical-scavenging capacity was presented as the extract concentration necessary to scavenge 50% of free radicals, IC_50_ (mg/mL).

#### 2.4.3. FRAP Method

The ferric-reducing antioxidant activity of bilberry samples was also investigated [30]. Namely, the FRAP reagent consisted of 10 mmol/L TPTZ solution in 40 mmol/L HCl (2.5 mL), 20 mmol/L FeCl_3_ (2.5 mL), and 0.3 mol/L acetate buffer (pH 3.6, 25 mL) and was heated at 37 °C. In a volume of 200 µL of water and 40 µL of bilberry extract, 1800 µL of FRAP reagent was added. The mixture was left at 37 °C for 10 min. The values of absorbance were read at a wavelength of 593 nm. The calibration curve was obtained using FeSO_4_. The results were shown as µmol Fe^2+^ equivalents per g of dried herbal material.

#### 2.4.4. CUPRAC Method

The cupric-ion-reducing antioxidant activity of bilberry extracts was investigated as well [31]. Firstly, copper(II)-chloride dihydrate (0.0853 g) was mixed with water to prepare a cupric(II) ion solution (10^−2^ mol/mL). Ammonium acetate (19.27 g) was mixed with 250 mL of water to prepare a 1 mol/mL ammonium–acetate buffer, while 0.078 g of neocuproine was dissolved in methanol (50 mL) to prepare neocuproine solution (7.5 × 10^−3^ mol/mL). Subsequently, the extract (800 µL), 1 mL of the solution of cupric(II) ions, 1200 µL of ammonium–acetate buffer, and 1 mL of the solution of neocuproine were mixed. The values of absorbance were read after 30 min at a wavelength of 450 nm. The calibration curve was obtained using Trolox. The results were presented as µmol TE per g of dried herbal material.

All absorbance readings were performed in triplicate in the Shimadzu UV-1800 UV/Visible scanning spectrophotometer (Shimadzu, Kyoto, Japan).

### 2.5. Antimicrobial Analysis

The antibacterial and antifungal activities of four *V. myrtillus* extracts (the samples prepared in all tested extraction methods to obtain the highest TPC) were also examined. All used microorganisms were clinical samples taken from the Institute for the Application of Nuclear Energy, Zemun, Serbia (microbiology laboratory). The following microorganisms were used: bacterial strains, including *Staphylococcus aureus*, *Escherichia coli*, *Enterococcus faecalis*, *Pseudomonas aeruginosa*, *Proteus* spp., and *Klebsiella* spp., and fungal strain *Candida albicans*. The determination of antibacterial and antifungal capacity was performed in the disk diffusion assay [32]. Cultivation of bacteria and fungus was performed in an incubator (37 °C) in aerobic conditions using blood agar (24 h) and Sabouraud agar (48 h), respectively. The inoculums of bacteria and fungus with an optical density of 1.5 × 10^8^ colony-forming units/mL were transferred to Mueller–Hinton and Sabouraud agar, respectively. Lyophilized extract dissolved in ultrapure water (175 mg/mL, 75 μL) was added to the wells of agar. The samples were kept in an incubator (37 °C) in aerobic conditions for 24 h. The criterion for the determination of the inhibitory potential of the extracts was the diameter of the inhibition zone (<0.5 cm—very weak inhibition, ≥0.5 cm—weak inhibition, ≥1 cm—medium inhibition, ≥1.5 cm—strong inhibition, and ≥1.8 cm—very strong inhibition). Each sample was tested in triplicate. Standard antibiotics for antibiogram (ampicillin, amoxicillin, amoxicillin with clavulanic acid, cephalexin, ceftriaxone, gentamicin, amikacin, nitrofurantoin, ciprofloxacin, trimethoprim, and fosfomycin) for bacteria and fluconazole for fungus were employed as a positive control with the aim to confirm the sensitivity of the examined microorganisms.

### 2.6. Determination of the Cell Viability

The potential cytotoxic effect of four bilberry extracts on the HaCaT cell line was investigated employing the MTT method described by Mosmann [33]. The keratinocytes (2 × 10^4^ cells/well) were seeded in 96-well plates with the complete medium (100 µL) and adhered in an incubator (37 °C, 5% CO_2_). Keratinocytes were rinsed after 24 h using a warm and sterile solution of PBS. Then, the cells were cultured in the complete medium using control or selected lyophilized *V. myrtillus* leaf waste extracts (at concentrations of 10 and 100 µg/mL) and left in an incubator (37 °C, 5% CO_2_) for 24 h. Later, the complete medium was eliminated, and keratinocytes were rinsed using a warm and sterile solution of PBS. Subsequently, PBS with 10% FBS (100 µL) and 0.5 mg/mL of MTT were added, and the keratinocytes were left in an incubator (37 °C, 5% CO_2_) for 2 h. Then, 10% SDS (0.01 N HCl, 100 μL) was transferred to each well and the samples were left in an incubator (37 °C, 5% CO_2_) for 24 h to provide solubilization of formazan crystals. After 24 h, the absorbance values were read at 570 nm in a microplate reader (ELx800, BioTek, Winooski, VT, USA).

### 2.7. Anti-Inflammatory Activity Analysis

The standard assays employed for the in vitro anti-inflammatory evaluation of *V. myrtillus* leaf waste extracts are described in the next sections.

#### 2.7.1. Cell-Based ELISA

The prospective in vitro anti-inflammatory potential of *V. myrtillus* extracts was investigated using the HaCaT cell line (ATCC) and a cell-based enzyme-linked immunosorbent assay (ELISA) [34]. Keratinocytes (2 × 10^4^ cells/well) were seeded in 96-well plates in 100 μL of DMEM/F12 cell culture medium with 10% FBS and 1% antibiotic and antimycotic solution. The samples were incubated overnight in a humified atmosphere with 5% CO_2_ (37 °C) for 24 h. Later, the medium was eliminated and fresh medium in the presence of *V. myrtillus* extracts at 10 and 100 μg/mL or in the absence of the extract (control) was added and left for 24 h. The next day, the samples were rinsed using a warm and sterile solution of PBS and treated with 1 μg/mL of bacterial LPS (1 h) to induce the production of proinflammatory cytokine IL-6. Then, the cells were rinsed using a warm and sterile solution of PBS, dried, and fixed using ice-cold acetone and methyl alcohol.

Staining for IL-6 was performed using the following procedure: the cells were rehydrated using a sterile solution of PBS, while the activity of endogenous peroxidase was stopped in the presence of 0.3% H_2_O_2_ (in the dark, 25 °C) for 30 min. Then, the keratinocytes were rinsed using a sterile solution of PBS, blocked using 1% BSA/PBS for 30 min (37 °C), treated using goat anti-human IL-6, and left in a humidified chamber (4 °C) for 24 h. Later, the samples were rinsed using a sterile solution of PBS, and biotinylated anti-goat IgG secondary antibodies were added for 30 min, followed by rinsing with PBS and incubation with ABC for another 30 min (in the dark, 25 °C). The reaction was visualized by the TMB solution (50 μL) and stopped with H_2_SO_4_ (0.2 M, 50 μL, 100 µL/well). The values of absorbance were measured at a wavelength of 450 nm using a microplate reader.

#### 2.7.2. Red Blood Cell (RBC) Membrane Stabilization Assay

Intending to investigate the anti-inflammatory potential in in vitro conditions, the RBC membrane stabilization method is employed as well [35,36]. Namely, the membranes of erythrocytes are similar to the lysosomal membrane, thus, erythrocytes were used to evaluate the impact of bilberry waste extracts on the membrane stability. Blood was taken from the Institute for the Application of Nuclear Energy, INEP, Zemun, Serbia (biochemical laboratory). Sodium oxalate was employed with the aim of preventing clotting. Blood was kept in a refrigerator for 24 h and centrifuged at a speed of 2500 rpm for 5 min using a Thermo Scientific Sorvall WX Ultra series ultracentrifuge (Thermo Fisher Scientific, Waltham, MA, USA). Sterile 0.9% *w*/*v* NaCl was employed to wash cells and the cells were centrifuged again. The mentioned procedure was repeated three times, and the cell volume was determined. The cells were mixed with PBS (10 mM, pH 7.4) to achieve a 40% suspension (*v*/*v*). Spectrophotometric measurement at a wavelength of 560 nm was used to estimate hemoglobin content in the suspension. Diclofenac (75 μg/mL) was a positive control, and the anti-inflammatory potential was expressed as the inhibition of the RBC lysis in the percentages.

Heat-induced lysis of red blood cells. The isotonic buffer with 50, 100, or 250 μg/mL of bilberry leaf waste extract (5 mL) was transferred to four centrifuge tubes. The same volume of pure isotonic buffer was transferred to another centrifuge tube (control). Then, erythrocyte suspension (50 μL) was added to each centrifuge tube with slight mixing. Two centrifuge tubes were left in a water bath (54 °C) for 20 min, while two centrifuge tubes were left in an ice bath (0–5 °C) for 20 min. The samples were centrifuged at a speed of 5000 rpm for 5 min. The values of absorbance of the supernatant were determined at a wavelength of 560 nm. The inhibition of lysis of RBC was determined using the following equation:

(5) $$\%\;\mathrm{inhibition}\;\mathrm{of}\;\mathrm{RBC}\;\mathrm{lysis}=100\;\times \;(1-\frac{OD2-OD1}{\mathrm{OD}3-\mathrm{OD}1})$$

OD_1_ is an unheated test sample, OD_2_ is a heated test sample, and OD_3_ is a heated control sample.

Hypotonic-induced lysis of red blood cells. The erythrocyte suspension in a volume of 50 μL was added to the hypotonic solution (154 mM sodium chloride in 10 mM sodium phosphate solution, pH 7.4) with bilberry extract at 50, 100, and 250 μg/mL concentrations (5 mL). The control samples contained hypotonic solution and RBC suspension or isotonic solution and the same concentrations of the extract. The samples were incubated at room temperature for 10 min and centrifugation was performed at a speed of 5000 rpm for 5 min. The values of absorbance of the supernatant were determined at a wavelength of 560 nm. The inhibition of RBC lysis was determined using the following equation:

(6) $$\%\;\mathrm{inhibition}\;\mathrm{of}\;\mathrm{RBC}\;\mathrm{lysis}=100\;\times \;(1-\frac{OD2-OD1}{\mathrm{OD}3-\mathrm{OD}1})$$

OD_1_ is the test sample in the isotonic solution, OD_2_ is the test sample in the hypotonic solution, and OD_3_ is the control sample in the hypotonic solution.

### 2.8. Physical Characterization

#### 2.8.1. Extraction Yield

The extraction yield was expressed as the dry matter content of the selected *V. myrtillus* extracts (the samples with the highest polyphenol content from all used extraction procedures) and determined according to the following equation:

extraction yield = 100 − ((a − b) × 100)/m(7)

*a*—weight of the dish with sample before drying, *b*—weight of the dish with sample after drying at 105 °C for 2 h (Memmert 30−1060, Memmert GmbH, Schwabach, Germany), and *m*—weight of sample. Extraction yield is presented in %.

#### 2.8.2. Measurement of the Conductivity

Measurement of the extract conductivity (the same extracts as in the measurement of the extraction yield, Section 2.8.1) was performed in a Zetasizer Nano ZS (Malvern Instruments Ltd., Malvern, UK). Each extract conductivity was determined in triplicate at 25 °C.

#### 2.8.3. Determination of Density, Surface Tension, and Viscosity

Density and surface tension analyses of four bilberry extracts (the same extracts as in the measurement of the extraction yield and conductivity) were performed in a Force Tensiometer—K20 (KRÜSS, Hamburg, Germany). Each sample in a volume of 20 mL was investigated in triplicate at 25 °C.

The extract viscosity was measured in the IKA Rotation Viscometer (Rotavisc *lo-vi*, IKA, Staufen, Germany). Each sample in a volume of 6.7 mL was measured in triplicate at 25 °C.

### 2.9. Statistics

The statistical analysis within the determination of optimal conditions in polyphenol extraction of *V. myrtillus* leaves was performed using statistical tools within the statistical software STATISTICA 7.0: one-way ANOVA, i.e., analysis of variance, Duncan’s *post hoc* test, and 2^3^ experimental design (three factors at the two most promising levels). In the factorial design, a 2-level screening design (Box, Hunter, and Hunter) has been conducted for the determination of the optimal conditions during the extraction process to prepare the sample with the highest polyphenol concentration. The results in figures and tables are shown as mean ± standard deviation and the difference was statistically significant when the *p* value was lower than 0.05 at n = 3. In the first screening, the impact of all examined factors (plant material:medium ratio, type of extraction medium, and period of extraction) on the TPC was determined. Statistical significance among various parameter levels has been estimated on the samples (n = 3) via analysis of variance and Duncan’s *post hoc* test (*p* < 0.05). Thus, mean values ± standard deviation with various letters in tables and figures statistically significantly differ. Subsequently, the choice of two promising levels of three tested factors (the levels that provided the highest polyphenol yield) was performed for the future 2^3^ experimental design (full factorial design). Analysis of variance followed by Duncan’s *post hoc* test was employed to investigate the effect of all examined factors on the extract antioxidant capacity and the effect of different procedures of the extraction on the chemical profile and physical properties, as well as biological potential, such as antioxidant and anti-inflammatory (RBC membrane stabilization assay) capacities of the extracts. Experimental design, as a statistical tool, was employed to optimize extraction factors, i.e., independent variables to obtain the conditions to reach the highest content of polyphenols. Namely, 2^3^ full factorial design (three extraction factors at the two most promising levels according to the first screening) was used to examine the effect and select the optimal plant material:medium ratio (1), extraction medium (2), and period of the extraction (3) to provide the extract with the highest TPC (a dependent variable).

Multivariate statistical analysis, i.e., principal component analysis (PCA), was conducted (STATISTICA 6.0 software) for the comparison of polyphenol compositions of the dried ethanol extracts (obtained in LC-MS analysis) according to the previous work published by Milutinović et al. [27]. The best output ordination in PCA based on the Bray–Curtis pairwise distance matrix was achieved using coded values of the compounds’ absolute content (g/100 g of dried extract).

Analysis of variance followed by a Tukey *post hoc* test (α = 0.05) was employed for the statistical analysis of the results obtained in the cytotoxicity assay (data passed the normality test). For cell-based ELISA, the Kruskal–Wallis test with Dunn’s *post hoc* test (α = 0.05) was performed. The difference was significantly different at *p* < 0.05. Statistical analysis was peformed using the statistical program GraphPad Prism 6.0 from GraphPad Software, Inc. (La Jolla, CA, USA).

Statistical analysis was not performed for the results of the antimicrobial activity test; thus, the values are not shown as mean ± standard deviation. The “stricter criteria” were used, represented in the analysis of antimicrobial potential. The determination of the antimicrobial capacity of the extracts was performed in three repetitions; the lowest value of the inhibition zone was taken as the result.

## 3. Results

The effects of the ratio between plant material and extraction medium (1:10, 1:20, and 1:30 g/mL), type of the extraction medium (water and 50 and 70% ethanol), period of exposure to the extraction solvent (30, 45, and 60 min for the extraction at room temperature, 15, 30, and 45 min for the extraction at 80 °C, 5, 15, and 30 min for the extraction by the ultrasound probe, and 1, 2, and 3 min for the extraction in a microwave reactor), heating, and ultrasound and microwave irradiation on the concentration of polyphenol compounds were investigated using analysis of variance followed by Duncan’s test within the software STATISTICA 7.0. The same tools have been useful in investigating the influence of plant material:extraction medium ratio, extraction solvent, period, and procedure on the radical-scavenging and ion-reducing activities of obtained extracts measured using ABTS, DPPH, FRAP, and CUPRAC tests. Regarding data related to the TPC obtained in the analysis of variance, as well as in Duncan’s test, two levels of three examined parameters were chosen for the following 2^3^ full factorial design (three extraction factors at two levels) to determine the conditions of the extraction that provide the extract with the highest content of polyphenol compounds. The next level of the study was the physicochemical characterization and investigation of antimicrobial and anti-inflammatory potential and impact on skin cells of four *V. myrtillus* extracts prepared in all tested extraction procedures for reaching the highest TPC.

### 3.1. Influence of Plant Material:Medium Ratio, Extraction Medium, and Period on Extracted Polyphenols

Since the extraction of polyphenols is significantly affected by the parameters and their levels during the extraction [37], preliminary screening of the parameters and their levels was carried out employing analysis of variance and Duncan’s test. Thus, the influence of plant material:extraction medium ratio, extraction solvent, and period on the TPC in *V. myrtillus* extracts was examined. The data are presented in Table 2.

As can be seen, the ratio between plant matrix and medium, as well as the type of the extraction medium, showed a significant effect on the TPC in all used procedures, while period of the exposure showed a significant influence on the polyphenol yield in maceration and UAE (Table 2). An increase in the ratio between herbal material and medium, from 1:10 g/mL to 1:30 g/mL, caused an increment of the TPC in *V. myrtillus* extracts (Table 2). Since polyphenol yield had the highest value using a ratio of 1:30 g/mL, followed by a ratio of 1:20 g/mL, these levels were included in the following experimental design. *V. myrtillus* ethanol extracts possessed statistically significantly higher TPC compared to aqueous extracts (Table 2). However, different ethanol concentrations (50% and 70%) did not significantly influence polyphenol content. Ethyl alcohol containing various amounts of water is widely employed for the isolation of polyphenols from plenty of herbal sources [11,38,39]. Since 50 and 70% ethanol *V. myrtillus* extracts showed the highest polyphenol yield, the mentioned mediums were subjected to the following experimental design. Additionally, the maximal polyphenol content was achieved after 45 min of maceration, 15 min of heating and sonication, and 2 min of MAE, while the extracts prepared after 30 min of maceration and 5 min of sonication possessed lower contents of polyphenol compounds (Table 2). However, in the extraction at 80 °C, the data indicated that there were no significant differences among various periods of exposure to the extraction solvent. In the preliminary screening, the polyphenol yield did not change after 60 min of extraction at elevated temperature. Regarding TPC results from MAE, the irradiation period showed a significant effect on polyphenol yield. Hence, the content of polyphenols achieved the highest value after 2 min of MAE. Since the maximal polyphenol content was reached after 45 min of maceration, while there were no statistically significant differences among 45 min and 60 min macerations, as well as due to reduced costs, periods of 30 and 45 min have been chosen for the experimental design. Furthermore, 15 and 30 min of heating and 5 and 15 min in UAE have been selected for the experimental design. In MAE, 2 and 3 min were used for further experimental design because the sample obtained at the 1st minute possessed statistically significantly lower polyphenol content.

### 3.2. The Influence of Procedures on Extracted Polyphenol Compounds

The effect of procedures (maceration, HAE, UAE, and MAE) on the polyphenol yield was also determined and the data are shown in Table 2 (for all examined factor levels) and Figure S1 (graphical charts for the polyphenol concentration in all samples obtained in four tested techniques).

Figure S1 shows that there was no significant difference in polyphenol yield between the extracts obtained using all four employed extraction techniques. However, it can be noticed that TPC was significantly higher in a 15 min extraction at 80 °C than in a 30 min maceration (Table 2). Nevertheless, there was no significant difference among the samples prepared in HAE for all exposure periods and the extracts prepared using 45 and 60 min of maceration (Table 2). Although ultrasounds can initiate significant degradation of cells and cell structure, and a higher recovery of polyphenol components for a shorter extraction time (the same TPC was found after 5 min of UAE and 30 min of maceration, Table 2), there was no significant difference among the samples made using prolonged maceration and UAE, as well as between the parallels (the same ratio and extraction medium) obtained in maceration, extraction at high temperature, and UAE (Table 2, Figure S1). As can be seen from Figure S1, the polyphenol content was not higher in the samples from MAE compared to other employed extraction procedures. Nevertheless, the TPC was the same after 2 min of MAE, 45 min extraction at room temperature, and 15 min of heating or sonication (Table 2).

### 3.3. Experimental Design (Full Factorial Design)

**Figure 1:**
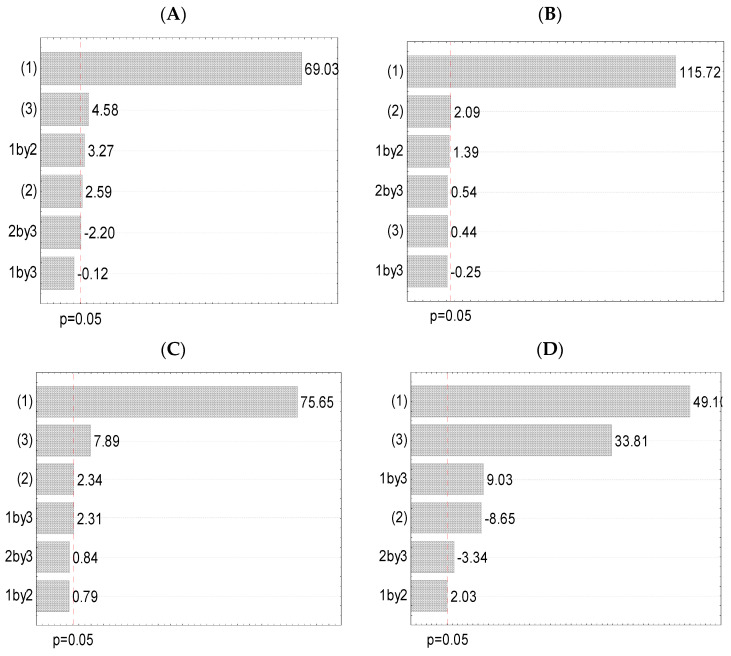
Graphical representation of Pareto charts of factors at two levels and interaction influence on the polyphenol content of *Vaccinium myrtillus* leaf dust extracts prepared (**A**) at room temperature, (**B**) at 80 °C, (**C**) by the ultrasound probe, and (**D**) in a microwave reactor; plant material:medium ratio, 1:20 g/mL and 1:30 g/mL (1), extraction medium, 50% and 70% ethyl alcohol (2), and exposure period, 30 and 45 min of extraction at room temperature, 15 and 30 min of heating, 5 and 15 min of sonication, 2 and 3 min of microwave irradiation (3); the difference was significant if *p* < 0.05.

A full factorial design was used to obtain the optimal extraction conditions (specific for each employed extraction procedure) with the aim of preparing the extracts with the highest polyphenol yield. Following the results of the first screening (Section 3.1), two levels of each examined factor, the ratio of plant material:medium (1:20 g/mL and 1:30 g/mL), extraction medium (50% and 70% of ethyl alcohol), and extraction period (30 min and 45 min maceration, 15 and 30 min of heating, 5 and 15 min of sonication, and 2 and 3 min of microwave irradiation), were used for the following 2^3^ experimental design. Two levels for each factor and the interactions between the factors are shown in Figure 1 as Pareto charts (the level of significance was *p* < 0.05). The effect estimates, as well as corresponding regression coefficients of factors and their interactions, were also determined, and the results are shown in Table S1. The measured and predicted values of the polyphenol yield, as the data from the experimental design, are also presented (Table 3).

A statistical tool, i.e., 2^3^ experimental design, was applied to examine the effect of the factors of the extraction process (independent variables) on the polyphenol concentration (dependent variable). Therefore, Figure 1 with Pareto graphs shows all observed parameters (at the two most promising levels) that can impact the TPC in every used technique. The Pareto charts show which factors (at two levels) and factor interactions possessed a significant impact on the polyphenol concentration in the samples.

Figure 1A and Table S1 show that the ratio between plant material and medium (factor 1) was the most relevant factor for the release of phenolic compounds from *V. myrtillus* leaf waste in maceration, followed by period (factor 3) and interaction among ratio and solvent type. The extraction medium (factor 2) and the interaction between extraction medium and period had lower, but also relevant, influence (Figure 1A and Table S1). However, the effect of the interaction between the ratio and period was not significant. Ćujić et al. [40] have also reported that the ratio between material and solvent and period were relevant parameters in reaching the highest TPC in the process of maceration.

Further, it can be noticed that only the ratio (factor 1) showed a significant influence on the TPC in the extracts from HAE. In contrast, all other observed parameters and their interactions did not show a statistically significant effect on the extracted amount of polyphenolics (Figure 1B and Table S1). Luthria [41] has reported that the ratio between herbal matrix and solvent significantly affected polyphenol release using extraction at high temperatures.

The plant material:medium ratio (factor 1) was the most dominant factor for the TPC using UAE, followed by period (factor 3) (Figure 1C and Table S1). Solvent type (factor 2) and interaction among ratio and period possessed a lower, but significant, influence, while the impact of the interactions among extraction medium and time and ratio and extraction solvent did not significantly affect the polyphenol yield. According to Paz et al. [42], the ratio and type of the extraction solvent were significant factors that affected the UAE of polyphenol compounds. Additionally, Ćujić et al. [40] have pointed out that the exposure period possessed a significant influence on the phenolic yield using UAE.

It can be noticed from Figure 1D and Table S1 that in MAE, the ratio among matrix and solvent (factor 1) was the most relevant factor in the extraction of polyphenols, followed by period (factor 3). The interaction among ratio and period, extraction medium (factor 2) and interaction among extraction medium and period showed a lower, but significant, effect on TPC. The impact of the interaction among the ratio and extraction solvent was insignificant, as in the case of the UAE. Regarding the study of Jovanović et al. [15], ratio, extraction medium, and irradiation time statistically significantly influenced polyphenol yield in the plant extracts obtained in MAE.

According to the results in Figure 1 and Table S1, the ratio was the most relevant parameter for polyphenol concentration in *V. myrtillus* leaf dust extracts in all four employed extraction techniques. It possessed a positive value, therefore, the upper level (a ratio of 1:30 g/mL in this study) resulted in a higher TPC. The solvent type represents the second dominant parameter to obtain the highest polyphenol yield in HAE and the third factor in UAE, whereas, in maceration and MAE, it was the fourth most important factor. Additionally, in MAE, it possessed a negative value meaning that a lower level (50% ethyl alcohol in this study) provides better efficiency, i.e., higher polyphenol yield. The extraction period was the second significant parameter for polyphenol concentration in the extraction at room temperature, UAE, and MAE, while in HAE, it was not significant. In the maceration, the interactions among ratio and extraction solvent, and extraction solvent and period, were relevant (as the third and fifth factors, respectively), whereas, in HAE, the influence of all interactions was not decisive. The presence of significant interaction among parameters shows that the influence of one parameter (e.g., the ratio between matrix and solvent) was not the same at all used levels of another factor (e.g., extraction time). In UAE, the interaction among the ratio and time was significant (as the fourth factor), while in MAE, the significant interactions were between ratio and period, and extraction medium and period (as the third and fifth factors, respectively).

The data from the 2^3^ experimental design shown as the measured and predicted mean values of the TPC are presented in Table 3.

The highest polyphenol content (measured mean values) in maceration was obtained at the highest ratio among matrix and medium, i.e., 1:30 g/mL, employing 50% ethyl alcohol as an extraction medium for 45 min (55.80 ± 0.23 mg GAE/g). The statistical analysis predicted the highest phenolic yield using the same levels of factors (56.02 mg GAE/g). The highest TPC (measured mean values) of the samples obtained from HAE was observed at a ratio of 1:30 g/mL using 50% ethyl alcohol during 30 min of heating (55.93 ± 0.11 mg GAE/g). The highest predicted value was under the same conditions (56.07 mg GAE/g). In UAE, the highest phenolic yield (measured mean values) was achieved using a ratio of 1:30 g/mL and 70% ethyl alcohol during 15 min of sonication (55.90 ± 0.30 mg GAE/g). The highest polyphenol content as a predicted mean was obtained using the same operational conditions (55.73 mg GAE/g). In MAE, the highest TPC (measured mean values) was reached at a ratio of 1:30 g/mL and employing 50% ethanol for 2 min in a microwave reactor (56.48 ± 0.15 mg GAE/g). The analysis predicted the maximum phenolic yield under the same extraction conditions (56.10 mg GAE/g). The difference between measured and predicted means was lower (<1%). Hence, a 2^3^ full factorial design can be a suitable model for the determination of the optimal extraction conditions for polyphenols of *V. myrtillus* leaf by-product.

Therefore, it can be concluded that regarding data from the 2^3^ experimental design, the extraction conditions to obtain *V. myrtillus* leaf waste extracts with the highest polyphenol concentration are a 1:30 g/mL plant material:extraction medium ratio, 50% ethanol as the solvent, and 2 min of extraction in a microwave reactor.

### 3.4. Total Flavonoids, Condensed Tannins, and Total Proteins of the Extracts

**Figure 2:**
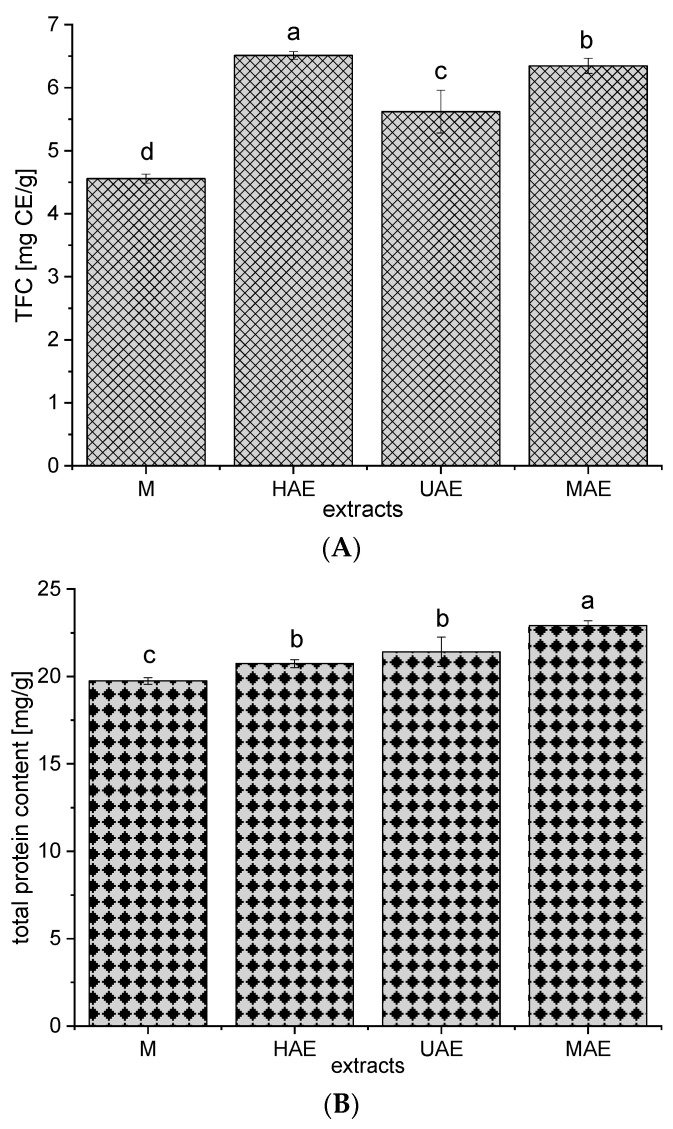

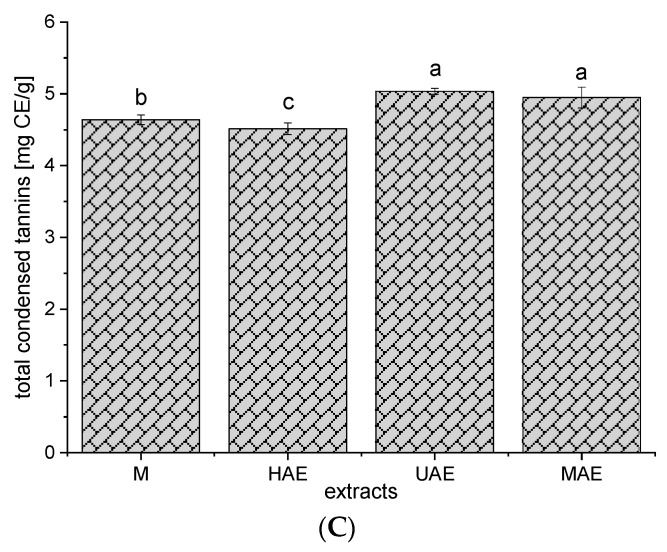
The concentrations of total flavonoids (**A**), condensed tannins (**B**), and total proteins (**C**) in *Vaccinium myrtillus* leaf by-product extracts obtained in maceration and heat, ultrasound, and microwave extractions (M, HAE, UAE, and MAE, respectively); mean values with different letters showed significant difference (*p* < 0.05; n = 3; analysis of variance, Duncan’s *post hoc* test); TFC, total flavonoid content; CE, catechin equivalent.

The selected samples (with the highest TPC using all tested procedures) were also analyzed in terms of the concentration of total flavonoids, condensed tannins, and total proteins. The data are graphically shown in Figure 2.

The highest flavonoid yield was determined in the sample from HAE (6.51 ± 0.06 mg CE/g), followed by the sample from MAE (6.35 ± 0.12 mg CE/g), while the macerate possessed the lowest flavonoid content (4.56 ± 0.07 mg CE/g) (Figure 2A). The TFC of the sample from UAE was statistically significantly higher (5.62 ± 0.34 mg CE/g) only in comparison to maceration. The concentration of condensed tannins was in a narrow range (4.51–5.04 mg CE/g) and achieved the highest yield in the samples from UAE and MAE (Figure 2B). According to the results presented in Figure 2C, the highest protein content was determined in the sample from microwave extraction (22.92 ± 0.27 mg/g), followed by heat and ultrasound extractions (20.74 ± 0.23 and 21.22 ± 0.70 mg/g, respectively). At the same time, the macerate possessed the lowest protein yield (19.74 ± 0.19 mg/g).

### 3.5. FT-IR Spectra of the Extracts

**Figure 3:**
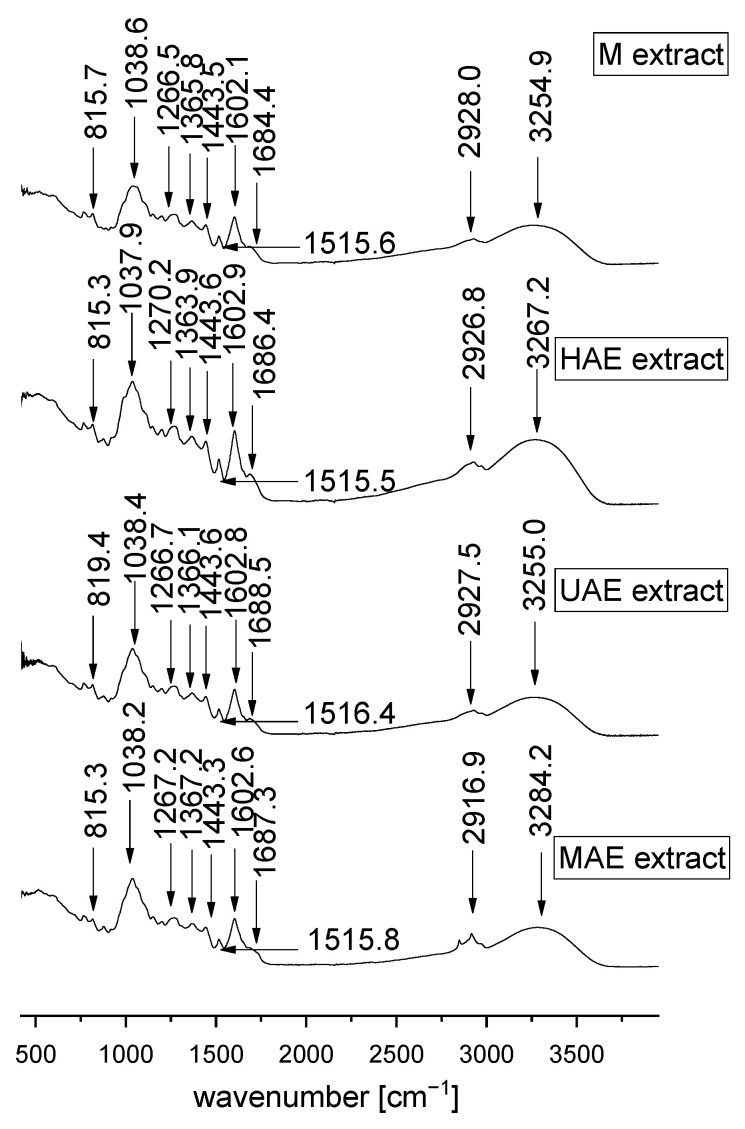
Fourier transform infrared (FT-IR) spectroscopy of selected lyophilized *Vaccinium myrtillus* extracts from leaf dust obtained in maceration and heat, ultrasound, and microwave extractions (M, HAE, UAE, and MAE, respectively).

FT-IR spectroscopy is a well-established technique for sample analysis, quality control, and information about the composition of plant extracts. Namely, a change in the absorption modes indicates changes in the sample composition or the presence of contamination [43]. FT-IR spectroscopy was employed to identify functional groups and investigate the impact of the used procedures for the extraction on the chemical properties of four *V. myrtillus* leaf extracts. FT-IR spectra of the extracts are shown in Figure 3.

FT-IR spectra of all tested *V. myrtillus* extracts (Figure 3) possess one wide peak from ~3000–3500 cm^−1^ (stretching vibrations of the OH groups, i.e., O-H stretching and H– bonds in alcohols and polyphenols) [44,45]. The mode at ~3300–2500 cm^−1^ may be O-H stretching, i.e., carboxylic acids [46]. The mode at ~2920 cm^−1^ may be associated with C-H stretching vibrations in the methyl/methylene groups corresponding to the vibrations which were shown by Belščak-Cvitanović et al. [47] and Jovanović et al. [48] who dealt with green tea and wild thyme extracts, respectively. The weak band at around 1680 cm^−1^ originates from the C=O stretching (secondary and tertiary amides), whereas the band at ~1600 cm^−1^ may be associated with carboxylic functional groups, as well as C-C stretching in aromatic rings present in plenty of compounds synthesized in secondary metabolism in plants, as well as in polysaccharide compounds [44,46,49]. The band at ~1500 cm^−1^ may be related to flavonoid compounds [50]. The mode in the range of 1500 to 1400 cm^−1^ correlated to C-C stretching (in-ring), i.e., aromatics, while the mode around 1440 cm^−1^ is related to C-H bending, i.e., alkanes (methyl group) [46]. The mode around ~1365 cm^−1^ may be related to the OH bending in alcohol and phenol functional groups, as well as N-O symmetric stretching of nitro components [44,46]. The band at ~1265 cm^−1^ originates from polyphenols, specifically C–C–O vibrations, while the mode at ~1200 cm^−1^ is attributed to C-N stretching, i.e., the functional group of aliphatic amines [46]. A class of overlapping bands in a range from 1200 cm^−1^ to 800 cm^−1^, peaking at ~1038 cm^−1^, may be associated with various C–O, C–O–C, and C–C vibrations in alcohols, sugars, and acid compounds. The lower-intensity band at ~815 cm^−1^ can be attributed to monoterpene components, i.e., C-H vibrations in the mentioned compounds [46,49]. The FT-IR spectra of *V. myrtillus* leaf waste extracts confirmed the presence of various functional groups (e.g., alcohols, phenols, carboxylic acids, alkanes, amides, amines, and aromatics) and bond types of bioactive compounds at various frequencies, thus rationalizing their applications as preparations with health benefits. Additionally, there was no difference in the FT-IR spectra of bilberry leaf dust extracts prepared using four tested techniques for the extraction.

### 3.6. LC-MS Analysis of the Extracts

In order to examine the impact of different extraction techniques on the presence or absence and content of the bilberry polyphenols, the qualitative and quantitative LC-MS analysis was performed for the four selected *V. myrtillus* leaf waste extracts. The investigation included those with the highest polyphenol concentration from all four extraction techniques, i.e., maceration (extraction medium 50% ethanol; 45 min), HAE (50% ethanol; 30 min), UAE (70% ethanol; 15 min), and MAE (50% ethanol; 2 min); all four extracts were prepared at 1:30 g/mL. The results are presented in Table 4 (chromatographic and spectral data) and Table 5 (the absolute content of the polyphenols). The obtained chromatograms are supplemented in Figure S2.

#### 3.6.1. Qualitative LC-MS Analysis of the Extracts

In the investigated *V. myrtillus* leaf waste extracts, 29 phenolic compounds were detected. Analyzing their UV and MS spectra, the structure of six compounds (**1**–**6**) was assigned to caffeic acid derivatives, eight to catechin derivatives (**7**–**10**, **12**, **14**, and **18**), eight compounds were flavonoids (quercetin glycosides, **13**, **15**–**17**, **21**–**23**, and **29**), and seven were described as *p*-coumaric acid derivatives (**19**, **20**, and **24**–**28**). Using commercial standard compounds, eight extracts′ constituents were identified (**5**, **7**, **11**, **13**, **15**–**17**, and **23**) [51,52,53,54,55,56,57,58,59]. Spectral data of detected compounds are given in Table 4, and the structures of some of the detected compounds are shown in Figure S3. MS spectra (full scan) were recorded in ESI negative ion mode at two fragmentor voltages (100 and 250 V), e.g., at a higher voltage of 250 V to generate MS spectra that include signals not only of the deprotonated molecule [M-H]^−^ but the signals of fragment ions originating from different molecule residues’ loss and/or reactions.

**Caffeic acid derivatives.** UV spectra (Table 4) of compounds **1**–**6** point to the structure of hydroxycinnamic acid derivatives. Compounds **5** and **6** had the fragmentation pattern of caffeoylquinic acids: signal of the deprotonated molecule [M–H]^−^ at *m*/*z* 353 (caffeoylquinic acid), diagnostic fragment ions signals at *m*/*z* 191 (quinic acid), 179 (loss of quinic moiety), 161, 135, and, based on the elution order, it was concluded that these two compounds might be stereoisomers. Chlorogenic acid (**5**) was identified using the standard, while the structure of **6** was proposed as its *cis* isomer, isochlorogenic acid, according to Clifford et al. [51]. Chlorogenic acid was previously identified and quantified in different extracts of bilberry leaves [52,53,54,55,56,57], while caffeic acid derivatives with [M-H]^−^ at *m*/*z* 707 were previously detected in water extract of bilberry leaves [58].

**Catechin derivatives.** The structures of five epicatechin or catechin-based oligomeric flavanols (procyanidins) were assigned as a B-type dimer (**7**), trimers (**8** and **11**), and A-type trimer (**10**), as well as (epi)gallocatechin trimer (**9**), according to their fragmentation pathways: [M-H]^−^ at *m*/*z* 577, 865, 863, and 881, respectively, and characteristic fragmentation ions formed by quinone methide (QM) cleavage of the inter-flavanoid bond, as well as heterocyclic ring fission (HRF) and retro Diels–Alder (RDA) fission of the heterocyclic ring system subunits [59,60,61], e.g., ions corresponding to dimer units at *m*/*z* 577 (for B-type trimers), 575 (for A-type trimers), and 289 originating from the (epi)catechin unit [56]. Procyanidin (**7**), a B-type dimer, was identified as procyanidin B2, while B-type trimer **11** as procyanidin C1, using standard compounds. Compounds **12**, **14**, and **18** with deprotonated molecule signals in MS spectra at *m*/*z* 765 (**12** and **18**) and 535 (**14**) were characterized as (epi)catechin derivatives due to their fragment ion at *m*/*z* 289.

**Quercetin flavonoids.** Compounds **13**, **15**–**17**, **21**–**23**, and **29** had the same UV spectra which indicated the 3-substituted flavonol, i.e., quercetin structure [62], and their MS spectra contained the signal of the fragment ion at *m*/*z* 301 [M-glycoside moiety-H]^−^ corresponding to the aglycone, as well as signals of fragment ions originating from the aglycone RDA reactions [62,63,64]. Quercetin 3-*O*-rutinoside (**13**) with a deprotonated molecule [M-H]^−^ at *m*/*z* 609, quercetin 3-*O*-galactoside (**15**) at *m*/*z* 463, quercetin 3-*O*-glucoside (**16**) at *m*/*z* 463, quercetin 3-*O*-glucuronide (**17**) at *m*/*z* 477, and quercetin 3-*O*-rhamnoside (**23**) at *m*/*z* 447 were identified by comparing their chromatographic data (Rt) and UV and MS spectral data with the data obtained for the authentic standard compounds. Compounds **21** and **22** had fragmentation patterns corresponding to quercetin acetylexoside (with signals of [M-H]^−^ at *m*/*z* 505, fragment ions at *m*/*z* 463 and 301) and quercetin pentosylhexoside ([M-H]^−^ at *m*/*z* 579, fragment ions at *m*/*z* 447 and 301, generated by pentose loss of 132 Da and hexose loss of 162 Da). The structure of compound **29** was tentatively found as quercetin hydroxy-methylglutaryl deoxyhexoside due to its [M-H]^−^ at *m*/*z* 591 and fragment ions signals at *m*/*z* 489, 447, and 301, where the ion at *m*/*z* 447 was probably obtained by the loss of 3-hydroxy-3-methylglutaroyl units from the deoxyhexose moiety and that at *m*/*z* 489 by cleaving from a 3-hydroxy-3-methylglutaroyl unit compared to the fragmentation pathway of quercetin-3-*O*-(4″-(3-hydroxy-3-methylglutaryl))-α-rhamnoside that had been previously identified in lingonberry fruit and leaves [65]. Flavonoids similar to **16**, **17**, **21**, **23**, and **29** were detected before in *V. myrtillus* leaves and/or stems [8,52,53,54,55,56,57,66].

**Table 4:** Spectral data of the detected polyphenols in *Vaccinium myrtillus* leaf waste extracts.

N^o^ *	Rt, min	λ_max_, nm	MS Data (250 V)	Compound	Ref./ Standard ***
**1**	7.10	328	707 (100) ** [M-H]^−^, 513, 417, 343, 305, 191	Caffeic acid derivative	[58]
**2**	7.89	328	707 (100) [M-H]^−^, 513, 417, 343, 305, 191	Caffeic acid derivative	[58]
**3**	8.71	328	707 (100) [M-H]^−^, 643, 593, 441, 191	Caffeic acid derivative	[58]
**4**	9.55	328	707 (100) [M-H]^−^, 577, 513, 191	Caffeic acid derivative	[58]
**5**	5.71	328	353 [M-H]^−^, 191 (100), 161, 135	Chlorogenic acid	St.
**6**	6.69	328	353 [M-H]^−^, 191 (100), 179, 173, 161, 135	Isochrologenic acid	[51]
**7**	12.05	230, 278	577 (100) [M-H]^−^, 451, 425, 407, 381, 339, 289	Procyanidin B2	St.
**8**	12.99	230, 278	865 (100) [M-H]^−^, 713, 691, 603, 575, 289, 245, 221, 137, 123	Procyanidin B-type trimer	[58,59,60]
**9**	14.70	230, 278	881 (100) [M-H]^−^, 755, 575, 289, 191	(epi)Gallocatechin-(epi) catechin-(epi)catechin trimer	UV/MS
**10**	16.59	230, 278	863 (100) [M-H]^−^, 711, 575, 451, 411, 289	Procyanidin A-type trimer	[58,59,60]
**11**	16.87	230, 278	865 (100) [M-H]^−^, 713, 577, 575, 425, 289	Procyanidin C1	St.
**12**	20.02	230, 278	765 (100) [M-H]^−^, 739, 725, 588, 449, 289	Catechin derivative	UV/MS
**13**	20.55	256, 265sh, 301sh, 354	609 (100) [M-H]^−^, 503, 301, 271, 243	Quercetin 3-*O*-rutinoside (rutin)	St.
**14**	20.87	230, 278	535 (100) [M-H]^−^, 425, 371, 311, 289, 191, 163, 147, 119	Catechin derivative	UV/MS
**15**	21.41	256, 265sh, 301sh, 354	463 (100) [M-H]^−^, 425, 301, 301, 271, 255, 243, 151	Quercetin 3-*O*-galactoside (hyperoside)	St.
**16**	21.59	256, 265sh, 301sh, 354	463 (100) [M-H]^−^, 301, 285	Quercetin 3-*O*-glucoside (isoquercitrin)	St.
**17**	21.59	256, 265sh, 301sh, 354	477 (100) [M-H]^−^, 301, 271	Quercetin 3-*O*-glucuronide (miquelianin)	St.
**18**	19.90	230, 278	765 [M-H]^−^, 635, 551, 535 (100), 373, 311, 289, 191, 163	Catechin derivative	UV/MS
**19**	22.51	290, 306	573 [M-H]^−^, 411 (100), 163, 145, 117	*p*-Coumaroyl malonyldihexoside	[54,58]
**20**	23.01	290, 306	593 (100) [M-H]^−^, 409, 341, 163, 145, 117	*p*-Coumaroyl diacetylhexoside derivative	[54,58]
**21**	23.44	256, 265sh, 301sh, 354	505 [M-H]^−^, 463, 301 (100), 245, 271, 255, 243	Quercetin acetylhexoside	UV/MS
**22**	24.32	256, 265sh, 301sh, 354	579 (100) [M-H]^−^, 447, 301, 285, 271, 255, 243, 227	Quercetin pentosyldeoxyhexoside	UV/MS
**23**	24.45	256, 265sh, 301sh, 354	447 (100) [M-H]^−^, 445, 341, 301, 285, 271, 255, 243, 189	Quercetin 3-*O*-rhamnoside (quercitrin)	St.
**24**	24.79	286, 306	411 (100) [M-H]^−^, 341, 163, 145, 117	*p*-Coumaroyl malonylhexoside	[54,58]
**25**	25.15	286, 306	409 (100) [M-H]^−^, 357, 315, 307, 163, 145, 119	*p*-Coumaroyl diacetylhexoside	[54,58]
**26**	25.46	286, 306	411 (100) [M-H]^−^, 249, 231, 163, 146, 145, 119, 117	*p*-Coumaroyl malonylhexoside	[54,58]
**27**	25.90	286, 306	411 (100) [M-H]^−^, 249, 163, 145, 119, 117	*p*-Coumaroyl malonylhexoside	[54,58]
**28**	26.38	286, 306	823 (100) [M-H]^−^, 489, 441, 435, 411, 341	*p*-Coumaroyl malonylhexoside dimer	[54,58]
**29**	27.81	256, 265sh, 301sh, 354	591 (100) [M-H]^−^, 489, 447, 341, 301, 271, 243, 189	Quercetin hydroxy- methylglutaryl deoxyhexoside	[65]

* Assigned numbers to the detected compounds, based on elution order; Rt—retention time. ** Numbers in brackets refer to the relative abundances of the ions (%) at a fragmentor voltage of 250 V. *** The references for the compounds’ UV and MS data comparison are given in square brackets; St.—commercial authentic compounds used for the identification.

**Coumaric acid derivatives.** The UV spectra of compounds **19**, **20**, and **24**–**28** with absorption maximums at 286 and 306 nm resembled those of 4-*O*-acylated derivatives of *p*-coumaric acid. Their MS spectra contained [M-H]^−^ signal at *m*/*z* 579 (**19**), 593 (**20**), 411 (**24**, **26**, and **27**), and 823 (**28**) and, in addition, fragment ions at *m*/*z* 411 [M-hexose moiety-H]- in the case of *p*-coumaroyl malonyldihexoside (**20**) and *p*-coumaroyl diacetylhexoside derivative (**20**) and/or *m*/*z* 249 (neutral loss of 162 Da), *m*/*z* 163 (*p*-coumaric moiety), *m*/*z* 145, and *m*/*z* 119 corresponding to *p*-coumaroyl malonylhexosides (**24**, **26**, and **27**), which was in agreement with earlier identification of *p*-coumaroyl hexosides [58]. In the mass spectrum of *p*-coumaroyl diacetylhexoside (**25**), the diagnostic signal at *m*/*z* 163 was formed by losses of two acetyl units (each of 42 Da) and a hexose unit (162 Da). Compound **28** was characterized as a *p*-coumaroyl malonylhexoside dimer. Compounds with *m*/*z* 411 have already been detected in *V. myrtillus* leaf extracts [8,54,58].

#### 3.6.2. Quantitative LC-MS Analysis of the Extracts

Caffeic acid derivatives, catechin derivatives, flavonoids, and *p*-coumaric acid derivatives detected in the dried ethanol extracts of *V. myrtillus* leaves were quantified using DAD peak areas (at 280 nm for procyanidins, 320 nm for phenolic acids, and 350 nm for flavonoids, except for flavonoids **16** and **17**). Because of the co-elution of quercetin 3-*O*-glucoside (**16**) and quercetin 3-*O*-glucuronide (**17**), for the quantification of these two flavonoids, the SIM data obtained for their deprotonated molecules [M–H]^−^ at *m*/*z* 463 (**16**) and *m*/*z* 477 (**17**) were used. Seven identified polyphenols, chlorogenic acid (**5**), procyanidin B2 (**7**), quercetin 3-*O*-rutinoside (**13**), quercetin 3-*O*-galactase (**15**), quercetin 3-*O*-glucoside (**16**), quercetin 3-*O*-glucuronide (**17**), and quercetin 3-*O*-rhamnoside (**23**), were quantified by the external standard method using identical commercial standards. The amounts of the other 16 detected constituents were determined using caffeic acid, chlorogenic acid, procyanidin B2, quercetin 3-*O*-glucoside, quercetin 3-*O*-rhamnoside, and *p*-coumaric acid, based on structural similarities. The regression equations, correlation coefficients (r^2^), linear ranges, LODs, and LOQs are given in Table 1. The results of the quantification (calculated as g/100 g of dried extract) are presented in Table 5.

**Table 5:** The quantities of constituents (**1**–**8**, **10**, **11**, **13**, **15**–**17**, **19**, **21**–**27**, and **29**) of selected *Vaccinium myrtillus* leaf waste extracts from maceration and heat, ultrasound, and microwave extractions (M, HAE, UAE, and MAE, respectively).

Compounds	Extracts			
	M	HAE	UAE	MAE
	Quantity ** (%, g of compound/100 g of dried extract)			
1 *	0.003 ± 0.000**	0.004 ± 0.000	0.002 ± 0.000	0.003 ± 0.000
2 ***	0.022 ± 0.007	0.046 ± 0.007	0.027 ± 0.004	0.020 ± 0.002
**3**	0.023 ± 0.003	0.017 ± 0.001	0.019 ± 0.000	0.023 ± 0.000
**4**	0.020 ± 0.000	0.033 ± 0.003	0.015 ± 0.000	0.021 ± 0.001
**5**	6.963 ± 0.039	12.661 ± 0.108	4.967 ± 0.016	6.439 ± 0.017
**6**	0.152 ± 0.008	0.255 ± 0.009	0.106 ± 0.003	0.128 ± 0.006
**7**	0.040 ± 0.000	0.560 ± 0.050	0.030 ± 0.000	0.008 ± 0.000
**8**	0.003 ± 0.000	0.004 ± 0.000	0.001 ± 0.000	0.002 ± 0.000
**9**	<LOQ	<LOQ	<LOQ	<LOQ
**10**	0.023 ± 0.006	0.011 ± 0.000	0.013 ± 0.004	0.009 ± 0.000
**11**	0.012 ± 0.000	0.021 ± 0.000	0.008 ± 0.000	0.007 ± 0.000
**12**	<LOQ	<LOQ	<LOQ	<LOQ
**13**	0.617 ± 0.020	1.132 ± 0.023	0.459 ± 0.011	0.584 ± 0.020
**14**	<LOQ	<LOQ	<LOQ	<LOQ
**15**	1.057 ± 0.014	2.060 ± 0.124	0.774 ± 0.031	0.987 ± 0.044
**16**	1.989 ± 0.057	3.545 ± 0.116	1.417 ± 0.038	1.399 ± 0.021
**17**	2.458 ± 0.008	4.707 ± 0.053	1.787 ± 0.009	2.068 ± 0.020
**18**	<LOQ	<LOQ	<LOQ	<LOQ
**19**	0.082 ± 0.007	0.151 ± 0.007	0.063 ± 0.004	0.082 ± 0.006
**20**	0.124 ± 0.010	0.184 ± 0.012	tr.	0.136 ± 0.008
**21**	0.559 ± 0.018	1.073 ± 0.020	0.426 ± 0.005	0.523 ± 0.011
**22**	0.188 ± 0.006	0.276 ± 0.025	0.144 ± 0.003	0.191 ± 0.007
**23**	0.440 ± 0.001	0.753 ± 0.076	0.312 ± 0.029	0.422 ± 0.034
**24**	0.005 ± 0.000	0.023 ± 0.009	0.011 ± 0.003	0.018 ± 0.006
**25**	0.230 ± 0.007	0.447 ± 0.011	0.171 ± 0.003	0.228 ± 0.068
**26**	0.028 ± 0.006	0.062 ± 0.001	0.023 ± 0.003	0.031 ± 0.004
**27**	0.547 ± 0.011	1.046 ± 0.020	0.407 ± 0.003	0.526 ± 0.008
**28**	tr.	tr.	tr.	tr.
**29**	0.081 ± 0.006	0.170 ± 0.010	0.065 ± 0.004	0.088 ± 0.006

* Assigned numbers to the detected compounds, based on elution order. ** The content is calculated as average of three different determinations ± standard deviation; < LOQ—below the limit of quantification; tr.—below 0.001%. *** Compounds **1**, **2**, **3**, and **4** were identified as caffeic acid; **6** as chlorogenic acid; **8**, **10**, and **11** as procyanidin B2; **21** as quercetin 3-*O*-glucoside; **22** and **29** as quercetin 3-*O*-rhamnoside; **19**, **20**, and **24**–**28** as *p*-coumaric acid. Compound names are given in Table 4.

**Figure 4:**
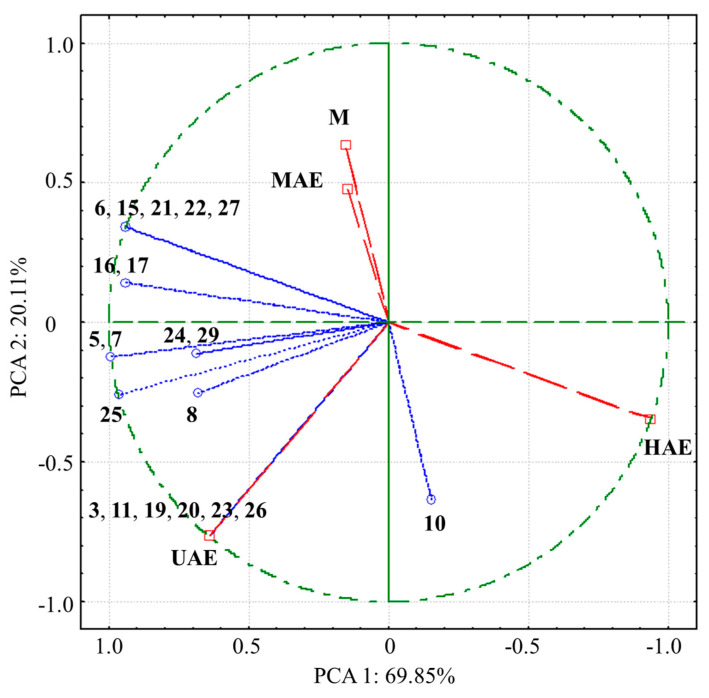
Principal component analysis (PCA) of the composition of *Vaccinium myrtillus* leaf waste extracts analyzed by LC-MS. Assigned numbers to the detected compounds and compound names are presented in Table 4. Empty dots are used for the quantified compounds; empty squares for the analyzed extracts from maceration and heat, ultrasound, and microwave extractions (M, HAE, UAE, and MAE, respectively).

In order to compare the analyzed extracts and find compounds that significantly contribute to differentiation between extracts, PCA was performed (Figure 4). Results showed that quantities of polyphenols (except **1**, **2**, **9**, **11**, **12**, **14**, **18**, and **28**) correlated with variance, contributing to special differentiation of the extract from UAE and the extract from HAE by the first axis (PCA1), as well as separation of these two from the extracts obtained using MAE and maceration, that were closely located in the fourth quadrant (Figure 4).

Compounds **5**–**7**, **22**, **25**, and **27** were highly positively correlated with factor 1 (0.82 to 0.99), contributing to it (69.85%). Compounds **3**, **19**, and **28** (-0.78) were highly negatively correlated with the second axis. Regarding absolute quantities of the compounds, chlorogenic acid (**5**: 12.661−4.967%) was the most abundant compound in all four extracts, followed by quercetin 3-*O*-glucuronide (**17**: 4.707−1.787%), quercetin 3-*O*-glucoside (**16**: 1.399−3.545%), quercetin 3-*O*-galactoside (**15**: 0.774−2.060%), and quercetin 3-*O*-rutinoside (**13**: 0.459–1.132%). The extract prepared using HAE showed the highest richness of almost all detected polyphenols, which was in accordance with the results of spectrophotometrically determined total flavonoid content (Section 3.4). Although the results of the 2^3^ full factorial design showed the MAE was the most suitable extraction technique to obtain the highest polyphenol yield (Section 3.3), HAE provided the extract with the highest yield of the individual phenolic compounds regarding the results of LC-MS analysis. It can be explained by the fact that microwaves can cause polymerization of individual polyphenol components. Namely, the mentioned polymers can be measured in the Folin–Ciocalteu assay, giving a higher TPC, while LC-MS analysis cannot quantify them. Chlorogenic acid (**5**: 4.967−12.661%), procyanidin B2 (**7**: 0.562–0.030%), quercetin 3-*O*-glucoside (**16**: 3.545−1.417%), quercetin 3-*O*-glucuronide (**17**: 4.707−1.787%), and quercetin derivative (**25**: 0.447–0.171%) exhibited significant quantitative divergence.

### 3.7. The Impact of the Parameters on the Antioxidant Capacity of Bilberry Extracts

The influence of various tested ratios between herbal matrix and medium (1:10 g/mL, 1:20 g/mL, and 1:30 g/mL), extraction mediums (water and 50% and 70% ethyl alcohol), and periods of the extraction (varied in all employed extraction techniques—maceration, HAE, UAE, and MAE) on the radical-scavenging and ion-reducing activities of *V. myrtillus* leaf waste extracts was investigated in different methods (ABTS and DPPH assays and FRAP and CUPRAC tests, respectively). The data are shown in Figure S4 (for ratio and extraction medium) and Table S2 (for exposure period). The effect of different techniques on the extract antioxidant activity is shown in Figure S5.

In all extraction techniques, the ratio possessed a significant influence on the antioxidant potential determined in four tests (Figure S4). The potential was lower as the extraction medium volume decreased, in the same manner as in the case of the TPC. Furthermore, the highest ABTS-radical-scavenging activity was achieved using 50% ethyl alcohol in four procedures for the extraction, but in HAE and UAE, there were no statistically significant differences among the extracts prepared using 50% and 70% ethyl alcohol (Figure S4A). Water extracts had the lowest activity, as in the case of polyphenol yield. According to the results of the DPPH method, there were no statistically significant differences among the extracts with 50% and 70% ethanol, while all water extracts possessed the highest IC_50_ value, i.e., the lowest DPPH-radical-scavenging potential (Figure S4B). FRAP assay shows that 50% ethanol samples possessed a higher ferric-ion-reducing activity compared to 70% ethyl alcohol parallels (except in the case of UAE where there was no statistically significant difference). Additionally, there was a difference between 70% ethanol and water extracts prepared using HAE (Figure S4C). According to the results of cupric-ion-reducing capacity, 50% and 70% ethanol extracts from HAE had the highest activity, while another 70% ethanol extract and all water extracts showed significantly lower values of reducing activity (Figure S4D).

In the ABTS assay, the exposure period significantly affected the antioxidant activity of bilberry samples prepared using UAE and MAE, where a shorter time gave the extracts with the lowest potential (Table S2). Nevertheless, the long extraction time in MAE (3 min) also caused a decrease in the ABTS-radical-scavenging capacity. The extraction period did not have an effect on the anti-DPPH activity (Table S2). The necessary period of extraction to obtain the samples with the highest ferric-ion-reducing potential in HAE and UAE was 30 and 15 min, respectively, while in maceration and MAE, the exposure period did not show a significant impact (Table S2). At the same time, the period necessary to prepare the samples with the highest cupric-ion-reducing capacity in maceration, UAE, and MAE was 60, 15, and 2 min, respectively. As can be seen from Table S2, in HAE, the extraction period did not have a significant influence on the cupric ion reduction activity of the extracts.

Figure S5A shows that the effect of four techniques for the extraction on the anti-ABTS capacity of the extracts has the following trend: HAE ≥ MAE ≥ maceration and UAE. However, DPPH, FRAP, and CUPRAC assays revealed that there were no significant differences in the antioxidant potential among the extracts obtained using different extraction methods (Figure S5B–D), in accordance with the TPC results (Figure S1).

### 3.8. Antimicrobial Activity of the Extracts

The antibacterial and antifungal activities of four bilberry leaf dust extracts were investigated; the data of the antibacterial activity towards two Gram-positive bacteria, *E. faecalis* and *S. aureus*, and four Gram-negative bacteria, *P. aeruginosa*, *E. coli*, *Klebsiella* spp., and *Proteus* spp., and antifungal activity towards *C. albicans* are presented in Table S3 (disk diffusion test).

*V. myrtillus* extracts possessed antibacterial potential towards *S. aureus* and *E. faecalis* (Table S3). All samples showed a very high inhibition effect on the growth of *S. aureus* (diameter of the inhibition zone ≥ 1.8 cm). The extracts prepared by heating and sonication had a strong inhibition impact on the growth of *E. faecalis* (inhibition zone ≥ 1.3 cm), while the samples obtained in maceration and MAE showed medium inhibition (inhibition zone ≥ 1.0 cm) on the mentioned bacteria. On the other hand, Gram-negative bacterial strains were resistant in the presence of the extracts prepared using *V. myrtillus* leaf waste (Table S3). Regarding the antifungal activity, all four extracts had no effect against *C. albicans* (Table S3).

### 3.9. The Effect of Bilberry Extracts on the Viability of HaCaT Cells

**Figure 5:**
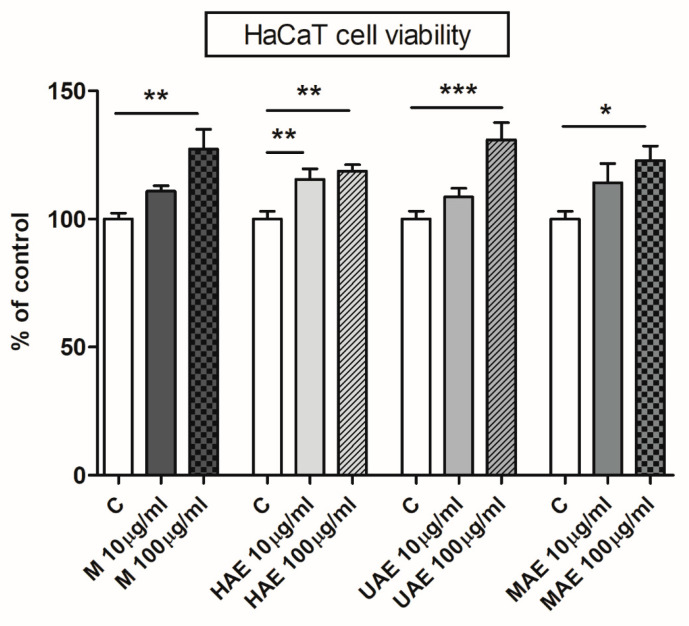
The effect of *Vaccinium myrtillus* extracts from maceration and heat, ultrasound, and microwave extractions (M, HAE, UAE, and MAE, respectively) on the viability of spontaneously immortalized keratinocytes; C, control (the keratinocytes in the absence of extracts); * *p* < 0.05, ** *p* < 0.01, and *** *p* < 0.001 (one-way analysis of variance and Tukey *post hoc* test).

In view of *V. myrtillus* application in folk medicine and its activities towards skin presented in different research [7,9], the impact of four bilberry extracts on keratinocyte viability was investigated. Figure 5 graphically shows the obtained data.

The potentially harmful effect of *V. myrtillus* samples was studied in vitro on spontaneously immortalized human keratinocytes, i.e., HaCaT cells. In the MTT assay, the absorbance of dissolved formazan is in correlation with the amount of viable keratinocytes. Nevertheless, the presence of plant extracts containing different components, as well as their interactions, represents a challenge in the screening methods with cell culture [67].

Figure 5 shows that the extracts had a significantly stimulating and dose-dependent effect on the viability of keratinocytes. Cell viability of keratinocytes was significantly increased following treatment with 100 µg/mL of the extracts to 127.3%, 118.7%, 130.9%, and 122.8% of control for the samples from the extractions at room and high temperatures, by the ultrasound probe, and in a microwave reactor, respectively. A lower concentration (10 µg/mL) had a significant effect on the cell viability only for the extract prepared using HAE (115.4% of control) (Figure 5).

### 3.10. In Vitro Anti-Inflammatory Effects of Bilberry Extracts

**Figure 6:**
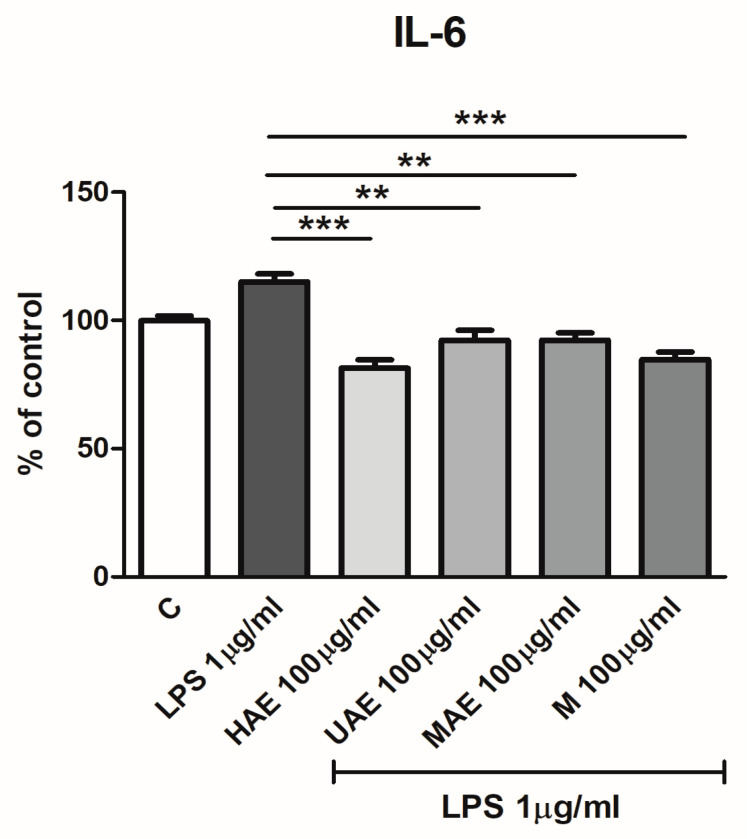
The effect of *Vaccinium myrtillus* extracts from maceration and heat, ultrasound, and microwave extractions (M, HAE, UAE, and MAE, respectively) on the bacterial lipopolysaccharide (LPS)-induced inflammation of spontaneously immortalized human keratinocytes (HaCaT cells); IL-6, interleukin 6; C, control (the cell without extract and LPS); ** *p* < 0.01, and *** *p* < 0.001 (Kruskal–Wallis and Dunn’s *post hoc* tests).

The in vitro anti-inflammatory potential of bilberry leaf extracts was investigated in (1) the model of inflammation of the HaCaT cells and release of IL-6 induced by bacterial LPS and (2) RBC membrane stabilization assay (lyses of erythrocytes induced by heat and hypotonic solution). The data are shown in Figure 6 and Table 6, respectively.

As can be seen in Figure 6, *V. myrtillus* extracts exerted an anti-inflammatory effect in LPS-treated HaCaT cells. Namely, pretreatment with extracts at 100 μg/mL had a significant inhibitory effect on LPS-induced production of proinflammatory cytokine IL-6. Production of IL-6 was 84.8%, 81.5%, 92.3%, and 92.2% of the control level for the extracts prepared at room and high temperatures and by the ultrasound probe and microwaves, respectively, and was lower than LPS-treated HaCaT cells’ IL-6 expression level by 35% (*p* < 0.001), 30% (*p* < 0.001), 25% (*p* < 0.01), and 25% (*p* < 0.01). A lower concentration of extracts (10 μg/mL) did not have a significant effect on IL-6 expression in the HaCaT cells.

RBC membrane stabilization which included heat- and hypotonic-induced lyses of erythrocytes was used as a mechanism for the investigation of in vitro anti-inflammatory potential of bilberry leaf waste extracts as well; the data are presented in Table 6.

*V. myrtillus* leaf extracts possessed promising concentration-dependent anti-inflammatory capacity in in vitro conditions (Table 6). In the case of heat-induced hemolysis, the extracts showed 81.5% to 83.9% inhibition of erythrocyte lysis at a 250 μg/mL concentration, while it was 70.6–74.6% at 100 μg/mL and 52.7–57.2% at 50 μg/mL. Nevertheless, there were no significant differences among the inhibition potential of the samples prepared in various techniques for the extraction at the highest employed concentration (250 μg/mL) probably due to the plateau. In hypotonic-induced hemolysis, in comparison to its heat-induced parallel, bilberry extracts possessed a lower potential of the inhibition of hemolysis only in the case of the lowest tested concentration (50 μg/mL) of the macerate and the samples from HAE and MAE processes. The level of the anti-inflammatory capacity varied in the range from 80.8% to 86.1% at 250 μg/mL, 71.3% to 78.5% at 100 μg/mL, and 49.8% to 57.4% at 50 μg/mL. However, in both types of hemolysis, diclofenac provided the highest percentage of red blood cell membrane stabilization.

### 3.11. Physical Characteristics of Bilberry Extracts

The values of the extraction efficiency presented as the extraction yield, electrolytic conductivity, density, surface tension, and viscosity of four *V. myrtillus* leaf extracts with the highest TPC in all used extraction procedures are presented in Table S4.

The percentage of the extracted compounds in *V. myrtillus* waste extracts obtained in all used techniques is shown in Table S4. The amount of the extractive substances depends on different factors, including extraction time, extraction solvent, matrix-to-medium ratio, the size of the herbal matrix, pH value, temperature, the presence of enzymes, etc. [68]. Table S4 shows that the procedure for the extraction significantly affected the extraction yield (MAE ≥ maceration ≥ UAE ≥ HAE). However, the amount of the extracted components was in a narrow range (1.14–1.94%).

The electrolytic conductivity was determined as the second property (Table S4). According to the literature, conductivity values can be a predictor of the extract’s antioxidant potential [69,70]. The conductivity of *V. myrtillus* leaf extracts varied from 0.18 ± 0.02 to 0.35 ± 0.03 mS/cm (Table S4) and, regarding the used extraction procedure, it follows the trend of MAE > maceration > HAE and UAE.

Due to further application and processes carried out on *V. myrtillus* leaf waste extracts, such as evaporation, drying, encapsulation, emulsification, or incorporation into various cosmetic, dermo-cosmetic, and pharmaceutical formulations, the rheological properties (density, surface tension, and viscosity) should be determined.

Table S4 shows that the density values of macerate and the extract from UAE were 0.961 ± 0.008 and 0.968 ± 0.019 g/cm^3^, respectively, whereas the extracts prepared using MAE and HAE have shown significantly lower density, 0.930 ± 0.009 and 0.919 ± 0.006 g/cm^3^, respectively. The surface tension of the extracts follows the trend UAE ≥ maceration ≥ HAE and MAE (Table S4). The surface tension varied in the range of 26.1 to 28.5 mN/m. The viscosity of all tested extracts was not significantly different and amounted to 2.99–3.19 mPa·s (Table S4).

## 4. Discussion

In the case of bilberry leaf extracts, the decrease in the ratio between herbal material and solvent resulted in a decrease in the polyphenol yield. According to the literature, polyphenol content increases with increased solvent volume that can dissolve a higher content of polyphenol compounds, preventing the saturation of the medium [65]. In the case of a large quantity of herbal matrix (using a ratio of 1:10 g/mL), faster saturation of the extraction solvent occurs, as well as increased medium viscosity and consequently the limitation of the activity of ultrasound waves and microwaves, therefore reducing the recovery of polyphenol [11,71]. The data on the effect of the type of extraction medium on the TPC (higher values for ethanol extracts in comparison to water parallels) indicate that polyphenol compounds from *V. myrtillus* leaves are preferentially released using water/ethanol mixtures. Namely, molecules of water in the organic medium make more polar surroundings for the extraction and changes in the hydrogen bonds, as well as cause the swelling of the herbal matrix, therefore resulting in higher efficiency of the extraction [72]. At the same time, ethanol can provide a lower dielectric constant of the solvent for extraction, molecules’ separation, and the facilitated diffusion of polyphenols [38,39]. Earlier research reported that the highest polyphenolic yield from various herbal materials was provided using different mixtures of water and ethanol as the surroundings for the extraction [73,74,75]. Additionally, the highest TPC levels were obtained using 50 and 75% alcohol for the extraction from the leaves of another *Ericaceae* species, *Gaultheria procumbens* L. [76]. Since Fick’s second law shows how the diffusion process results in a concentration change over time, prolonged extraction time is expected to result in the enhancement of polyphenol concentration in the extracts [75,77]. Additionally, the extraction period, i.e., exposure time, is a function of the weight of desired components and the weight of ballast substances, thus the mentioned parameter should be optimized for every extraction technique and every plant material [10,75]. However, in HAE, the data showed that there were no significant differences among different extraction times. The reason may lie in the presence of two levels in the release of polyphenolics: (1) an increase in the TPC over 15 min, and (2) significantly slower polyphenol release for 60 min [75]. In our preliminary screening, polyphenol yield did not change after 60 min of HAE. Friedman et al. [78] and Vergara-Salinas et al. [79] showed that extended extraction time could result in a decrease in polyphenol content due to the sensitivity of phenolic components, especially during heating, as well as the occurrence of their degradation, oxidation, and polymerization. In the case of MAE, 1 min was too little time for a complete polyphenol release, while a longer irradiation time (3 min) can destroy polyphenols, as well as cause the excessive recovery of proteins, lipids, and carbohydrates as ballast compounds [11,80], therefore, the TPC was the highest at the intermediate extraction time used (2 min). In addition, polyphenol yield was statistically significantly better in the sample obtained in HAE (15 min) in comparison to the macerate obtained after 30 min, which can be explained by the mechanism of HAE. Namely, a high temperature improves extraction efficiency by disruption of plant cells, and causes higher polyphenol release, by the increase in polyphenol solubility and, consequently, the increase in their mass transfer and by the decrease in extraction medium surface tension and viscosity, accelerating the extraction process [11,79,81,82]. The absence of a significant difference among the samples obtained after a prolonged time of maceration and extraction by the ultrasound probe may lie in the disadvantages of the application of sonication for the recovery of bioactives. According to the literature, apart from the mentioned advantages, ultrasound waves can degrade polyphenol antioxidant components by the generation of free radicals, thus decreasing the TPC values, as well as the extract’s antioxidant properties [10,11,83]. Consequently, UAE can be a successful and faster technique for the recovery of polyphenol components of *V. myrtillus* leaf waste, but it was not better (for the extracts prepared after 15 min) in comparison to 45 min maceration and 15 min HAE. Furthermore, polyphenol yield was the same after 2 min of MAE, 45 min maceration, and 15 min of heating and sonication, which can be explained by the mechanism of microwaves. Microwaves cause the significant and uniform warming of the surroundings for the extraction, superheating of the cellular matrix, disruption of the cellular structures, and hence a higher polyphenol recovery in a shorter extraction time [11,83]. Comparing all used extraction techniques with respect to the industrial demands, including high polyphenol content and reduced extraction time, MAE can be a suitable method for the recovery of *V. myrtillus* leaf waste polyphenols. Since it was proven that the mentioned extract possessed the highest content of polyphenols, future experiments should be focused on the investigation of potentially harmful compounds in the final extract from bilberry leaf waste, such as allergens, heavy metals, or impurities, with the intention of obtaining an active and safe ingredient for pharmacological, dermo-cosmetic, and cosmetic formulations.

The flavonoid yield was statistically significantly higher in the extract obtained by the ultrasound probe in comparison to the macerate. Namely, the presented data agreed with the previous findings, where the best flavonoid yield was achieved in UAE, whereas lower flavonoid concentration was determined in the extracts made in a conventional extraction procedure [84]. However, a lower value of flavonoid concentration obtained in UAE compared to HAE and MAE can imply that flavonoids are more sensitive to ultrasound waves than to elevated temperatures. These findings are favorable for future applications of high temperature or microwaves in *V. myrtillus* leaf waste extraction, particularly for flavonoid-rich samples that can exert various biological activities. Namely, according to the literature data, most flavonoid compounds show, among others, antioxidant, i.e., free-radical-scavenging activity, anti-inflammatory, and anticancer properties, whereas some flavonoids possess antiviral potential [85].

The obtained levels of tannin concentrations in this research are lower compared to the literature results for the extracts of bilberry leaves (0.8–6.7%) [86]. However, the same study has shown that the concentrations of tannins significantly depended on geographical origin and the effect of environmental stress which can explain the difference between our results and literature data. Lower tannin concentration measured in the extract prepared using HAE cannot be related to the higher temperature because a thermogravimetric study showed high thermal resistance of tannins [87] and the sample from MAE possessed significantly higher tannin content even at the temperature of 100 °C (Figure 2B). Since tannins are located in plant cell vacuoles and can form complexes with proteins [88], mechanical effects, including ultrasound and microwaves, are probably necessary for their release in the extraction medium [11,83]. Thus, the thermal effect alone (present in the HAE method) is not sufficient to release a higher amount of tannins. Tannins represent an antiphotoaging compound that possesses antiwrinkle and antioxidant properties, as well as can prevent damage caused by UVB irradiation [89]. They also show various antimicrobial, antiparasitic, and anti-inflammatory properties [90].

The highest protein content was determined in the bilberry leaf sample prepared in MAE, whereas the lowest value was measured in macerate. The obtained results are expected since the destructive effects of microwaves on plant tissues and cells cause higher recovery of proteins in the extraction surroundings which was shown in several studies [15,80,91,92]. Lin et al. [93] suggested that plant proteins are promising agents for skin regeneration. However, sometimes proteins in plant extract can represent ballast substances causing only an increase in the total extraction yield, and they can be allergens, while the content of target compounds can be lower. Thus, the selection of the extraction procedure depends on the target active components.

In view of the previously published results of quantitative analysis of hydroethanol extracts from *V. myrtillus* leaves or leaves with twigs/stems, differences in the amounts of each compound were found in herbal drugs from different locations. The qualitative and/or quantitative profile of the extracts can also be influenced by the extraction conditions [4]. Thus, the amounts of the four most abundant constituents of maceration, HAE, UAE, and MAE are either comparable to or higher than the amounts previously reported for some of the extracts studied [52,53,54,55,56,57]. For example, the content of chlorogenic acid (6.09–8.70%) in four different extracts prepared by maceration or ultrasound extraction (twigs and leaves from the Cindrel Mountains, Batrana Peak, Romania; extraction medium 50% ethanol) was comparable to the content in the macerate and UAE extracts studied in this work, in contrast to the content of quercetin 3-*O*-rutinoside (3.23–4.66%), which was up to 4-fold higher [52]. The UAE extract prepared via sonotrode (leaves from organic cultivation in Huelva, Spain; 30% ethanol) showed a higher content of chlorogenic acid (9.07%) compared to the UAE extract in this research, while quercetin 3-*O*-glucuronide was only present at 0.73% [53]. In the other UAE extract (leaves with stems from the local market in Tuzla, Bosnia and Herzegovina; extraction with 80% ethanol), the highest amounts were found for quercetin 3-*O*-galactoside (4.44%) and chlorogenic acid (2.08%) [55], while for the percolate (leaves from the production area of the Institute for Medicinal Plant Research “Dr Josif Pančić”, Belgrade, Serbia; double percolation method of extraction, 50% ethanol), Kuzmanović Nedeljković et al. [56] have reported chlorogenic acid (5.40%) and quercetin 3-*O*-glucoside (4.02%) as the dominant compounds.

Plant extracts are specific in terms of the presence of various components apart from polyphenols, which can exert different biological activities. The interactions of extract constituents, synergism (when the combined activity of two compounds is higher than the sum of their effects when given separately), or even potentiation (when one compound does not elicit a response on its own but enhances the reaction, i.e., the effect, of another compound) have an important role in the bioactivity as well. Therefore, the focus of the research segment dealing with potential biological activities of bilberry leaf waste has been the investigation of the overall effect of all compounds present in the extracts.

*V. myrtillus* extract antioxidant activity shown in all four employed assays was better in ethanol samples compared to aqueous parallels. Literature data showed that herbal extracts prepared using ethanol possessed better antioxidant capacity than aqueous samples [81]. In addition, prolonged exposure to microwaves (3 min) resulted in a decrease in ABTS-radical-scavenging potential due to the degradation of sensitive antioxidant components. In contrast, time did not influence the DPPH-radical-scavenging potential of bilberry extracts, implying that the compounds responsible for the mentioned activity are released in the first phase of extraction and remain stable even after a longer extraction process. In the FRAP assay, the extraction time significantly influenced extract antioxidant potential for the samples prepared using HAE and UAE, whereas it was not the case in maceration and MAE. However, according to the literature data, long periods of sonication can result in decreased antioxidant ability because of the chemical breakdown of phenolic components, such as flavonoids and anthocyanins [94]. At the same time, in the FRAP test, the exposure period significantly affected the ferric-reducing antioxidant potential of the extracts obtained at room temperature, by ultrasound probe, and in a microwave reactor, but not in HAE. The differences between the samples that show the highest antioxidant capacity in the different assays used may lie in the fact that different metabolites besides phenolics or their derivates and their interactions can influence plant extract antioxidant capacity [95]. Nevertheless, in the DPPH, FRAP, and CUPRAC assays, there was no significant difference in the activity among the samples obtained in different extraction procedures that followed data on the polyphenol yield. The absence of a statistically significant difference in the antioxidant property between the samples prepared using various procedures for the extraction is not surprising. Namely, despite the excessive release of active and non-active compounds into extraction medium during the use of ultrasound waves or microwaves, there is also the destruction of sensitive molecules with antioxidant properties by ultrasound and microwave irradiation and free radical production by the ultrasound probe. Since the extract’s anti-DPPH activity can be similar despite variations in polyphenol amount, it can be explained by the chemical makeup of polyphenol antioxidants as well as the concentration of individual polyphenols that affect the capacity of the sample to neutralize DPPH radicals. Additionally, synergistic and antagonistic interactions between flavonoid compounds also have an important role in the overall antioxidant potential [96]. Chlorogenic acid exhibits antioxidant capacity via hydrogen donation in the DPPH, ABTS, and FRAP assays [97,98,99]. Namely, Xu et al. [98] and Wu [99] reported that the mentioned acid possessed strong ABTS- and DPPH-radical-scavenging activity, as well as a higher efficiency in reducing Fe^3+^ to Fe^2+^, due to the higher number of available hydroxyl groups. The flavonoids rutin, hyperoside, isoquercitrin, and miquelianin identified in the bilberry leaf waste extracts also exert antioxidant activities by performing direct radical-scavenging reactions (ABTS and DPPH radical), reducing Cu^2+^ and Fe^3+^, and/or reducing lipid peroxidation by increasing superoxide dismutase (SOD) and catalase activity [100,101,102,103,104,105,106]. In ABTS and DPPH assays, all four flavonol heterosides showed strong and concentration-dependent scavenging activity (over 90% at 100 ppm; in some tests IC_50_ ≈ 2 μM) [100,101,106]. Moreover, it was observed that rutin can prevent oxidative stress by inhibiting reactive oxygen species, nitric oxide, and malondialdehyde secretion, increasing SOD activity, and restoring glutathione peroxidase activity in vitro in the HaCaT cell model (treated with H_2_O_2_) [102]. Apak et al. [107] showed that the polyphenol concentration determined using the Folin–Ciocalteu assay was in correlation with extract antioxidant activity determined using the CUPRAC tests. Additionally, polyphenol compounds that possessed the highest values in the CUPRAC assay according to the literature data were gallic, caffeic, and chlorogenic acids, rutin, quercetin, catechin, and epicatechin and its derivatives [107]. Hence, the LC-MS method confirmed the presence of most of the mentioned polyphenol compounds in *V. myrtillus* leaf waste extracts (Table 4). Furthermore, the absence of correlation among polyphenol content values and anti-ABTS capacity may lie in the presence of non-phenolic components that possess antioxidant properties, such as plant pigments, thiols, mannitol, glucose, free organic acids, D-ascorbic acid, and proteins [96]. According to the results of the antioxidant capacity observed in a comparison of the samples prepared in various employed techniques, the discrepancy between the four antioxidant tests is not surprising. Namely, oxidation probes, kinetics of the reactions, targeted compounds, and experimental conditions (pH values, wavelength for the measurements, incubation period, etc.) are different depending on the employed test.

Since the differences between the total polyphenol content of the four final extracts (prepared under the optimal conditions) varied in a very narrow range (from 55.80 to 56.48 mg GAE/g), all of them were investigated in terms of biological potential (antimicrobial and anti-inflammatory activities). Namely, as ultrasound and microwave extractions require expensive devices, the biological activities of the extracts prepared using simple extraction procedures, such as maceration and extraction at a high temperature, should also be examined. Additionally, since, apart from polyphenols, other bioactive compounds in plant extracts and their interactions can be responsible for the overall biological potential of the sample, it is necessary to notice if any of the tested extraction techniques particularly stand out to extract compounds that can increase some of the mentioned biological activities.

Phytocompounds possess direct biostatic/biocidal potential and the ability to increase the effects of antibiotics by increasing the permeability of bacteria walls [108]. Tested bilberry leaf extracts had antibacterial potential towards *E. faecalis* and *S. aureus*, while all tested Gram-negative bacterial strains were resistant. According to the literature data, the antibacterial capacity of bilberry leaves in water and ethanol was examined using *E. faecalis* and *E. coli*, and the samples showed a higher inhibition of the growth of *E. faecalis* [86]. Leaf extracts of different *Vaccinium* species showed high inhibitory effects toward *S. aureus* [108,109]. The literature data also showed higher sensitivity to polyphenols in the case of Gram-positive bacterial strains in comparison to Gram-negative, as in the case of *V. myrtillus* leaf waste extracts. Ștefănescu et al. [86] have reported that non-flavonoid compound and total polyphenol concentrations possessed a stronger correlation with the inhibitory influence on the growth of *S. aureus* in contrast to flavonoid content. Various mechanisms of polyphenol’s role in the inhibitory effects on bacterial growth were described, including membrane degradation, extracellular microbial enzyme inhibition, the disruption of bacterial metabolism, as well as deprivation of the components necessary for the growth of bacteria [110]. According to Vučić et al. [111], the most sensitive strain of bacteria towards ethanol extracts of *V. myrtillus* leaves was *E. faecalis*, while *E. coli* was the most resistant. Literature data reported that chlorogenic acid (present in all tested blueberry extracts in a higher amount according to the LC-MS analysis) showed an inhibitory effect on bacterial growth via destroying the membrane integrity, resulting in the release of bacteria metabolites, triggering bacteria cell inactivation, and disturbing the normal metabolic pathways of bacterial organisms [112]. In microdilution assay, chlorogenic acid (minimal inhibitory concentration (MIC) = 0.5–1 mg/mL; minimal bactericidal concentration (MBC) ≥ 1 mg/mL), rutin (MIC = 0.5–1 mg/mL; MBC = 0.5–1 mg/mL), and isoquercitrin (MIC = 0.25–1 mg/mL; MBC = 0.25–1 mg/mL) exhibited antibacterial activity toward *E*. *coli*, *P*. *aeruginosa*, and *K*. *pneumoniae* [113]. Also, in the cylinder–plate (diffusion) method, rutin and isoquercitrin inhibited the growth of *S*. *aureus* and/or *E*. *coli* and *P*. *aeruginosa* (10.2–16.1 mm) [114]. Hyperoside and quercetin showed moderate to low antibacterial activity toward *P. aeruginosa*, *E. coli*, and *S. aureus* [114,115]. Additionally, in the present study, all tested bilberry extracts did not affect *C. albicans*. The absence of anticandidal potential may lie in the fact that there is a lack of ellagitannins, which are the most dominant and potent antimicrobial components toward *Candida* species [116].

Deters et al. [117] and Werdin [118] have reported that secondary metabolites of plants, such as polyphenolics (tannins and proanthocyanidins, also quantified in *V. myrtillus* leaf waste extracts), have an important role against wound infection and stimulative effects on the proliferation of keratinocytes. The cellular system employed in the research represents a powerful tool because it can select extracts with stimulative effects on the making of new tissue and regeneration of a wound. Thus, herbs that are traditionally used or claimed to be potent in the treatment of ulcers or sores should have a positive impact in MTT assay on human keratinocytes [119]. The obtained results of extracts’ impact on cell viability can be regarded as encouraging because the extracts did not demonstrate harmful effects on keratinocytes with benefits related to radical-scavenging, ion-reducing, antimicrobial, and anti-inflammatory properties (presented in Section 3.7, Section 3.8 and Section 3.10), thus *V. myrtillus* extracts obtained from leaf by-products can be implemented in pharmacological, dermo-cosmetic, and cosmetic preparations for skin.

Regarding the literature, berry compounds from pomace, whole extracts, polyphenol-rich samples, and specific fractions of polyphenols can decrease gene expression for proinflammatory markers and their release in cell lines with LPS-induced inflammation [120,121,122]. Various plant metabolites in a dose-dependent manner can reduce the expression of genes and LPS-induced production of proinflammatory cytokines, such as IL-6 [121]. However, the anti-inflammatory potential is due to an array of components and their synergism rather than the polyphenol fraction from blueberry [120]. This can explain the similar anti-inflammatory potential of bilberry leaf waste extracts prepared using different extraction techniques in the LPS-induced inflammation of keratinocytes. Gu et al. [120] have shown that both phenolic and volatile compounds inhibited proinflammatory cytokine secretion, such as IL-6, in LPS-stimulated macrophages via suppression of the NF-κB signaling pathway. Nevertheless, quercetin (present in the tested bilberry leaf waste extracts as well) has been proven to moderately reduce IL-6 secretion in LPS-stimulated macrophages [123]. The LC-MS analysis showed that the most dominant compounds in tested bilberry extracts were chlorogenic acid, rutin, hyperoside, isoquercitrin, and miquelianin, and their anti-inflammatory activity in the LPS-induced inflammation was already documented. Namely, Kang et al. [124] and Kim et al. [125] evidenced the potential of chlorogenic acid and hyperoside in the reduction of the production of proinflammatory markers, such as TNF-α and IL-6, in monocyte/macrophage cell lines. Additionally, rutin, isoquercitrin, and miquelianin significantly decreased or limited the expression levels of IL-6, IL-1β, and TNF-α in the HaCaT cells which were up-regulated by UVB irradiation. These flavonoids showed no significant influence on HaCaT cell viability. However, the compounds significantly protected HaCaT cells from UVB-induced cell death in a concentration-dependent manner [102,106,126,127]. A comparison between the obtained values in the present study and the literature data is not possible because various bilberry extracts (different plant parts, extraction mediums, and extraction procedures) and cell lines were used. Additionally, Su et al. [122] showed that the extract’s influence on the production of IL-6 and inhibitory concentrations varied depending on the plant cultivar as well.

All tested bilberry leaf waste extracts showed in vitro anti-inflammatory potential on the heat- and hypotonic-induced RBC membrane lysis in a dose-dependent manner, i.e., the potential increased with an increase in the extract amount. Since the samples successfully inhibited the heat-induced lysis of erythrocytes, the data prove that stabilization of the erythrocyte membrane represents an additional mechanism of the anti-inflammatory activity of bilberry extract also mentioned in the literature data and traditional medicine [1,4,7]. Serum protein and fluid efflux in the surrounding tissue, as well as the increase in permeability of the membrane and release of the inflammatory intermediates, can be prevented by membrane stabilization. Thus, the investigation of the extract’s influence on the hypotonic-induced lysis has shown that *V. myrtillus* extracts can prevent the lysis of erythrocytes, i.e., provide the stabilization of membrane probably by disrupting the release of the enzymes responsible for the lysis of the cells and recovery of various mediators of the inflammatory process [35]. In addition, since triterpenoid and flavonoid compounds possess exceptional anti-inflammatory potential, plant extracts can provide RBC membrane stabilization. Hence, erythrocytes are similar to lysosomes, thus, the RBC membrane stabilization in the presence of the extracts suggests that they can stabilize lysosomal membranes as well [128]. Namely, activated neutrophils provide the release of the lysosomal constituents responsible for the inflammation of the tissue and consequently damage due to extracellular leakage. Therefore, the prevention of the lysosomal constituent recovery resulted in anti-inflammatory activity. The data of this study indicated that bilberry leaf extract compounds maintained the tonicity and balance of the erythrocyte membrane and thereby prevented its lysis. Bioactives of the plant extracts, including tannins, flavonoids, coumarins, etc., can have an important role in decreasing the plasma membrane stress caused by high temperature or hypotonic solution [129]. Bonarska-Kujawa et al. [130] reported that chlorogenic acid molecules are positioned mainly in the outer part of the erythrocyte membrane (hydrophilic region) and do not induce lysis or osmolality changes, preventing the diffusion of free radicals into the membrane interior. In addition, chlorogenic acid significantly prevented an increase in the osmotic fragility of erythrocytes, i.e., enhanced their resistance in hypotonic solution, showing an effective protective activity during the co-incubation treatment [131]. In addition, flavonoid compounds, including quercetin, rutin, and hyperoside, exerted in vitro stabilizing properties on the erythrocyte membrane against hypotonic-induced lysis [101,132].

The percentages of the extracted components from *V. myrtillus* leaf are in correlation with the content of total proteins in the extracts. The highest extraction yield of the extract from MAE did not correlate with its TPC. The reason may lie in the simultaneous extraction of other ballast substances (lipids, carbohydrates, organic acids, proteins, etc.) that are later presented in the final sample. In addition, waxes, phytosterols, and fatty oils, as non-polar or non-volatile substances, increase the value of the extraction yield as well [75,133]. The gradient of concentrations between the extraction medium and liquid within the plant particles can significantly affect the extraction yield. Therefore, the size of the herbal matrix, porosity, as well as surface contact among the medium and plant material particles influence the efficiency of the extraction process and consequently the extraction yield [134]. During microwave irradiation, excessive degradation of the herbal particles occurs, thus there is more contact among the extraction medium and matrix, and a higher content of the extracted substances can be observed.

The measured values of the extract conductivity agreed with the results presented in the literature [69,135]. Quite low conductivity of the examined *V. myrtillus* extracts (all prepared using a 1:30 g/mL ratio) may be due to the significant influence of the ratio between solvent and plant matrix on the conductivity. It is also shown by Čutović et al. [135] that the conductivity of plant extracts decreased with the increase in the ratio between the herbal material and extraction medium. The conductivity follows the trend microwave extraction > maceration > extractions using high temperature or ultrasounds. Since, in the literature, an extract with a higher antioxidant capacity possesses a higher conductivity, the antioxidant activity of four selected bilberry samples measured using ABTS- and DPPH-radical-scavenging assays and FRAP and CUPRAC methods was compared to their conductivity values. Regarding the data from the ABTS and DPPH assays for the mentioned four samples (31.4 ± 0.8 µmol TE/g and 1.81 ± 0.02 µg/mL for maceration, 48.3 ± 1.8 µmol TE/g and 1.89 ± 0.01 µg/mL for HAE, 29.6 ± 0.9 µmol TE/g and 1.80 ± 0.03 µg/mL for UAE, and 31.3 ± 0.9 µmol TE/g and 1.85 ± 0.05 µg/mL for MAE, respectively), it can be deduced that antioxidant activity did not correlate with the measured conductivity at all levels. According to the literature data, electrolytic conductivity is affected by extraneous ions and their species [64]. Therefore, the highest measured electrolytic conductivity in the extract obtained in MAE can be explained by the increment of the ions in the extraction medium. However, according to the results of the FRAP and CUPRAC assays for four bilberry samples (15.0 ± 0.2 µmol Fe^2+^/g and 42.9 ± 0.7 µmol TE/g for maceration, 14.5 ± 0.3 µmol Fe^2+^/g and 41.2 ± 0.8 µmol TE/g for HAE, 14.1 ± 0.5 µmol Fe^2+^/g and 41.3 ± 0.3 µmol TE/g for UAE, and 15.5 ± 0.2 µmol Fe^2+^/g and 46.5 ± 0.8 µmol TE/g for MAE, respectively), the antioxidant capacity correlated with the determined electrolytic conductivity values at all levels. Nevertheless, the conductivity of bilberry samples was significantly low (as in the case of drinking water), thus the differences between the examined samples cannot be taken into account for claims on the differences between the extracts’ antioxidant potential. Therefore, the determination of the antioxidant potential of the plant extracts employing established antioxidant tests is essential.

The literature data showed that the density of the extracts is in correlation with the extraction yield [136]. However, in the case of prepared *V. myrtillus* extracts, the density did not correlate with the extraction yield, probably because the values varied in a rather narrow range. The extraction medium can cause the adsorption of polyphenolic compounds at a lower value of the surface tension. Additionally, rapid penetration of the medium within the plant material leads to the enhancement of the surface between solid and solvent, as well as consequently to a higher release of phenolic components. However, low differences between the surface tension of the tested four extracts (3.9–8.1%) may, among others, be the reason for the absence of significant differences among the polyphenol yield values of the samples prepared using different extraction techniques (Figure S1), despite the potential of high temperature, ultrasound waves, and microwaves in comparison to the mixing as the only process that can allow better diffusion of polyphenols in the extraction at room temperature.

According to Oroian et al. [137], viscosity values are not in correlation with density, however, both mentioned variables depend on the temperature. Specifically, a higher temperature results in a decrease in density and viscosity. An earlier study [138] reported that viscosity is linearly correlated with surface tension, whereas surface tension is linearly correlated with density. In *V. myrtillus* extracts, the samples with a higher value of density (macerate and UAE) also possessed higher viscosity and surface tension values. Additionally, the constitution of the surrounding solvent for the extraction significantly affects extract viscosity. Namely, a higher amount of water provides surroundings for hydrolytic reactions by hydrolytic enzymes resulting in a lower value of viscosity because of the degradation of various viscous compounds [139].

## 5. Conclusions

The presented research was designed to investigate the influence of various ratios between herbal matrix and solvent, extraction mediums, and exposure periods on the polyphenol concentration of *V. myrtillus* leaf waste extracts obtained by employing extractions at room and high temperatures and ultrasound and microwave extractions. The data showed that the ratio was the most dominant parameter, followed by the exposure period (except in HAE). Concerning the 2^3^ experimental design, the optimal extraction conditions and procedure to achieve the highest phenolic yield were a ratio of 1:30 g/mL and 50% ethyl alcohol during 2 min microwave irradiation, i.e., MAE. The flavonoid content was significantly higher in the samples obtained at a high temperature, while the content of tannins was the highest after UAE and MAE. The highest total protein amount was determined in the sample obtained by the microwaves. The FT-IR spectra showed the functional groups of bioactives. LC-MS analysis revealed the presence of 29 phenolic components, while chlorogenic acid and quercetin 3-*O*-glucuronide are the most abundant components in bilberry extracts. The effect of various extraction conditions on the extracts’ antioxidant properties depended on the used method for the determination of the mentioned activity. All extracts showed a very strong inhibitory activity on *S. aureus*. The extracts prepared using HAE and UAE possessed a strong inhibition on the growth of *E. faecalis*, whereas the samples from maceration and MAE showed medium inhibition. Additionally, all extracts showed a stimulative effect on viability of keratinocytes and in vitro anti-inflammatory potential (proven in cell-based ELISA and erythrocyte stabilization assays). Bilberry extracts reduced the production of proinflammatory cytokine IL-6 induced by LPS and in a dose-dependent manner inhibited the heat- and hypotonic-induced RBC membrane lyses. The extract prepared in MAE possessed the highest yield of the extraction, while electrolytic conductivity, density, surface tension, and viscosity values varied in a narrow range. The study provides evidence of the optimal conditions and procedure to extract the highest TPC, chemical composition, antioxidant, antimicrobial, anti-inflammatory, and skin cell viability effects, and physical characteristics of *V. myrtillus* leaf waste extracts. The presented results can be regarded as encouraging because the extracts did not demonstrate harmful effects on keratinocytes in addition to the shown antioxidant, antimicrobial, and anti-inflammatory properties, therefore, the extracts can be incorporated in pharmaceutical, dermo-cosmetic, and cosmetic preparations. In future experiments, different fractions obtained from bilberry leaf waste can be developed with the aim of expanding the potential range of the extract application, such as tannin-rich extracts for potential wound healing and/or infection treatments or an ingredient of sun protection products, flavonoid-rich extracts for potential per os application in gastrointestinal disorders, or phenolic-acid-rich extracts as a potential ingredient of functional foods for the prevention of metabolic syndrome.

## Figures and Tables

**Table 1:** Regression equations, correlation coefficients (r^2^), linear ranges, and limits of detection (LOD) and quantification (LOQ) of reference compounds.

Compound	Regression Equations	*r* ^2^	Linear Range (μg/mL)	LOD (μg/mL)	LOQ (μg/mL)
Caffeic acid	y = 13,866.0695x − 2.1700	0.9999	0.07–1.75	0.022	0.066
Chlorogenic acid	y = 7359.9706x + 21.8506	0.9999	0.05–4.00	0.015	0.044
Procyanidin B2	SIM: y = 43,695,889.4939x + 227,090.6258	0.9995	0.08–0.80	0.043	0.132
Quercetin 3-*O*-galactoside	y = 2755.8034x + 22.2383	0.9999	0.02–4.00	0.010	0.060
Quercetin 7-*O*-glucoside	y = 3450.5525x + 19.4631	0.9999	0.07–4.00	0.020	0.103
Quercetin 3-*O*-glucoside	SIM: y = 11,180,730.8564x + 263,073.4506	0.9971	0.08–0.80	0.043	0.132
Quercetin 3-*O*-glucuronide	SIM: y = 24,101,774.7157x + 872,524.4889	0.9944	0.08–0.80	0.043	0.132
Quercetin 3-*O*-rhamnoside	y = 2941.0244x + 7.5674	0.9999	0.05–3.50	0.030	0.082
*p*-Coumaric acid	y = 16,845.1037x + 47.4654	0.9998	0.08–1.75	0.026	0.078

**Table 2:** The impact of parameter levels on the total polyphenol content (TPC) in *Vaccinium myrtillus* extracts from leaf by-product.

		TPC (mg Gallic Acid Equivalent/g of Dried Herbal Matrix)							
Parameter	Level	Extraction Procedures							
		Maceration		HAE		UAE		MAE	
Plant material:medium ratio (g/mL)	1:10	19.96 ± 0.70 ^c^*		20.01 ± 0.41 ^c^		20.44 ± 0.52 ^c^		19.26 ± 0.60 ^c^	
	1:20	38.32 ± 0.88 ^b^		37.92 ± 0.79 ^b^		38.27 ± 0.75 ^b^		38.57 ± 0.40 ^b^	
	1:30	54.64 ± 1.10 ^a^		55.65 ± 0.23 ^a^		55.62 ± 1.70 ^a^		56.48 ± 1.68 ^a^	
Extraction medium	50% EtOH	55.47 ± 1.28 ^a^		55.55 ± 0.35 ^a^		56.49 ± 0.40 ^a^		57.06 ± 1.90 ^a^	
	70% EtOH	55.71 ± 1.48 ^a^		56.10 ± 0.68 ^a^		56.04 ± 0.84 ^a^		56.26 ± 1.03 ^a^	
	Water	47.43 ± 1.73 ^b^		48.20 ± 1.93 ^b^		47.54 ± 1.68 ^b^		49.95 ± 0.98 ^b^	
Period ** (min)		30	53.37 ± 0.34 ^b^	15	56.37 ± 1.23 ^a^	5	53.79 ± 1.06 ^b^	1	41.55 ± 0.42 ^c^
		45	55.38 ± 0.51 ^a^	30	55.43 ± 1.32 ^a^	15	56.82 ± 1.17 ^a^	2	56.48 ± 1.68 ^a^
		60	55.16 ± 0.70 ^a^	45	55.47 ± 1.02 ^a^	30	56.73 ± 0.89 ^a^	3	49.95 ± 1.12 ^b^

* Values followed by different letters in each column (for every investigated parameter) showed statistically significant differences (*p* < 0.05; n = 3; analysis of variance, Duncan’s *post hoc* test). ** The period of extraction varied depending on the extraction procedures; EtOH, ethanol; HAE, extraction at 80 °C; UAE, extraction by the ultrasound probe; MAE, extraction in a microwave reactor. All three solid-to-solvent ratios were combined with all used extraction solvents and all employed extraction times in all tested extraction techniques.

**Table 3:** Full factorial design (2^3^) for the investigation of the parameters’ impact on the total polyphenol content (TPC) of *Vaccinium myrtillus* extracts from leaf dust obtained in maceration and heat, ultrasound, and microwave extractions with the measured and predicted means.

Ratio *	Extraction Medium	Period	Ratio (g/mL)	Extraction Medium	Period (min)	TPC (mg Gallic Acid Equivalents/g)	
						Maceration	
Design			Factor levels			Measured	Predicted
−1	−1	−1	1:20	50% EtOH	30	38.32 ± 0.62	38.09
−1	1	−1	1:20	70% EtOH	30	38.23 ± 0.25	38.45
**1**	**−1**	**1**	**1:30**	**50% EtOH**	**45**	**55.80 ± 0.23**	**56.02**
1	1	1	1:30	70% EtOH	45	55.38 ± 0.18	55.16
−1	−1	1	1:20	50% EtOH	45	39.50 ± 0.50	39.72
−1	1	1	1:20	70% EtOH	45	39.27 ± 0.30	39.05
1	−1	−1	1:30	50% EtOH	30	53.37 ± 0.24	53.59
1	1	−1	1:30	70% EtOH	30	54.71 ± 0.55	54.49
						**Heat extraction**	
						Measured	Predicted
−1	−1	−1	1:20	50% EtOH	15	37.92 ± 0.56	37.95
−1	−1	1	1:20	70% EtOH	15	38.00 ± 0.50	37.92
**−1**	**1**	**−1**	**1:30**	**50% EtOH**	**30**	**55.93 ± 0.11**	**56.07**
−1	1	1	1:30	70% EtOH	30	55.43 ± 0.22	55.45
1	−1	−1	1:20	50% EtOH	30	38.00 ± 0.20	37.97
1	−1	1	1:20	70% EtOH	30	38.13 ± 0.51	38.16
1	1	−1	1:30	50% EtOH	15	54.54 ± 0.16	54.51
1	1	1	1:30	70% EtOH	15	55.10 ± 0.48	54.96
						**Ultrasound extraction**	
						Measured	Predicted
−1	−1	−1	1:20	50% EtOH	5	36.87 ± 0.15	37.07
−1	1	−1	1:20	70% EtOH	5	37.43 ± 0.21	37.23
1	−1	1	1:30	50% EtOH	15	55.62 ± 1.20	55.82
**1**	**1**	**1**	**1:30**	**70% EtOH**	**15**	**55.90 ± 0.30**	**55.73**
−1	−1	1	1:20	50% EtOH	15	38.36 ± 0.41	38.15
−1	1	1	1:20	70% EtOH	15	38.50 ± 0.62	38.70
1	−1	−1	1:30	50% EtOH	5	53.88 ± 0.13	53.68
1	1	−1	1:30	70% EtOH	5	54.00 ± 0.10	54.20
						**Microwave extraction**	
						Measured	Predicted
−1	−1	−1	1:20	50% EtOH	3	41.00 ± 1.1	40.53
−1	−1	1	1:20	70% EtOH	3	37.83 ± 0.28	38.30
**−1**	**1**	**−1**	**1:30**	**50% EtOH**	**2**	**56.48 ± 0.15**	**56.10**
−1	1	1	1:30	70% EtOH	2	52.52 ± 0.42	52.99
1	−1	−1	1:20	50% EtOH	2	38.57 ± 0.30	39.04
1	−1	1	1:20	70% EtOH	2	35.27 ± 0.64	34.80
1	1	−1	1:30	50% EtOH	3	45.55 ± 0.32	45.02
1	1	1	1:30	70% EtOH	3	44.50 ± 0.54	44.03

* Ratio between herbal matrix and medium; EtOH, ethanol; the highest TPC and extraction conditions to achieve the mentioned values (for each extraction procedure) are bold.

**Table 6:** The impact of *Vaccinium myrtillus* samples obtained from leaf dust in maceration and heat, ultrasound, and microwave extractions (HAE, UAE, and MAE, respectively) on the erythrocyte membrane stabilization (heat- and hypotonic-induced lyses of erythrocytes).

Sample	Concentration (µg/mL)	Inhibition of Hemolysis at 54 °C (%, Mean ± SD)	Inhibition of Hemolysis in Hypotonic Solution (%, Mean ± SD)
Maceration	50	55.7 ± 1.8 ^f,1^*	51.9 ± 1.1 ^h,2^
	100	72.7 ± 0.7 ^c,1^	73.1 ± 1.7 ^ef,1^
	250	81.5 ± 1.5 ^b,1^	80.8 ± 1.2 ^c,1^
HAE	50	62.8 ± 1.0 ^e,1^	57.4 ± 0.6 ^g,2^
	100	74.6 ± 1.5 ^c,2^	78.5 ± 1.4 ^d,1^
	250	83.9 ± 1.7 ^b,2^	86.1 ± 1.0 ^b,1^
UAE	50	52.7 ± 1.1 ^g,1^	52.9 ± 0.9 ^h,1^
	100	70.6 ± 0.8 ^d,2^	75.9 ± 1.2 ^de,1^
	250	82.5 ± 1.2 ^b,2^	85.6 ± 1.4 ^b,1^
MAE	50	57.2 ± 1.0 ^f,1^	49.8 ± 1.5 ^h,2^
	100	72.6 ± 1.1 ^c,1^	71.3 ± 0.6 ^f,1^
	250	82.9 ± 1.0 ^b,1^	84.4 ± 1.8 ^b,1^
Diclofenac (control)	75	91.0 ± 0.5 ^a,2^	93.9 ± 0.8 ^a,1^

* Different letters (a–g) in each column and different numbers (1 or 2) between the columns represent the presence of significant differences based on analysis of variance and Duncan’s *post hoc* test (n = 3; *p* < 0.05).

## Data Availability

The datasets generated during and/or analyzed during the current study are available from the corresponding author upon reasonable request.

## Supplementary Materials

The following supporting information can be downloaded at: https://www.mdpi.com/article/10.3390/pharmaceutics16060740/s1, Figure S1: The influence of extraction procedures on total polyphenol content *Vaccinium myrtillus* leaf waste extracts from maceration and heat, ultrasound, and microwave extractions (HAE, UAE, and MAE, respectively); the same letter represents the absence of significant differences based on one-way analysis of variance and Duncan’s *post hoc* test at *p* < 0.05; TPC, total polyphenol content; GAE, gallic acid equivalent; Figure S2: Chromatograms of four *Vaccinium myrtillus* extracts obtained from leaf dust by maceration and heat, ultrasound, and microwave extractions (M, HAE, UAE, and MAE, respectively); numbers of compounds are given in Table 4; Figure S3: Structures of the components identified in four *Vaccinium myrtillus* extracts from leaf dust using the LC-MS method; numbers of compounds are presented in Table 4; Figure S4: The effect of solid-to-solvent ratio and solvent type on (A) ABTS and (B) DPPH neutralization, (C) ferric-ion-reducing potential, i.e., FRAP, and (D) cupric-ion-reducing activity, i.e., CUPRAC of *Vaccinium myrtillus* extracts from waste of the leaves prepared using maceration and heat, ultrasound, and microwave extractions (HAE, UAE, and MAE, respectively); different letters represent statistically significant difference between the parallels from the same extraction procedures based on one-way analysis of variance and Duncan’s *post hoc* test (n = 3; *p* < 0.05); TE, Trolox equivalent; Figure S5: The impact of extraction methods on (A) ABTS and (B) DPPH neutralization, (C) ferric-ion-reducing antioxidant power (FRAP), and (D) cupric-ion-reducing activity (CUPRAC) of all obtained *Vaccinium myrtillus* extracts from maceration and heat, ultrasound, and microwave extractions (HAE, UAE, and MAE, respectively); various letters mean different population groups based on one-way analysis of variance and Duncan’s *post hoc* test (n = 3; *p* < 0.05); TE, Trolox equivalents; IC_50_, the concentration of extract required to neutralize 50% of DPPH radicals; Table S1: The optimization of maceration and heat, ultrasound, and microwave extractions (HAE, UAE, and MAE, respectively) of *Vaccinium myrtillus* by-product from leaves employing as a statistical tool a 2^3^ experimental design; Table S2: The effect of exposure period on ABTS- and DPPH-radical-scavenging properties, ferric-ion-reducing potential, i.e., FRAP, and cupric-ion-reducing activity, i.e., CUPRAC of *Vaccinium myrtillus* extracts from maceration and heat, ultrasound, and microwave extractions (HAE, UAE, and MAE, respectively); Table S3: The antibacterial and antifungal potential of four *Vaccinium myrtillus* extracts obtained using leaf by-product and maceration and heat, ultrasound, and microwave extractions (HAE, UAE, and MAE, respectively) investigated in the disk diffusion assay; Table S4: The data of *Vaccinium myrtillus* leaf dust extracts’ extraction yield (EY), conductivity (G), density (ρ), surface tension (γ), and viscosity (η).
