# Supplementary material for: Vaccinium myrtillus L. Leaf Waste as a Source of Biologically Potent Compounds: Optimization of Polyphenol Extractions, Chemical Profile, and Biological Properties of the Extracts
Source: Pharmaceutics. 2024 May 30;16(6):740. doi: 10.3390/pharmaceutics16060740 (PMC11206553; doi:10.3390/pharmaceutics16060740)
Supplement: Supplementary file 1 [file pharmaceutics-16-00740-s001.zip › pharmaceutics-3014698-supplementary.pdf]

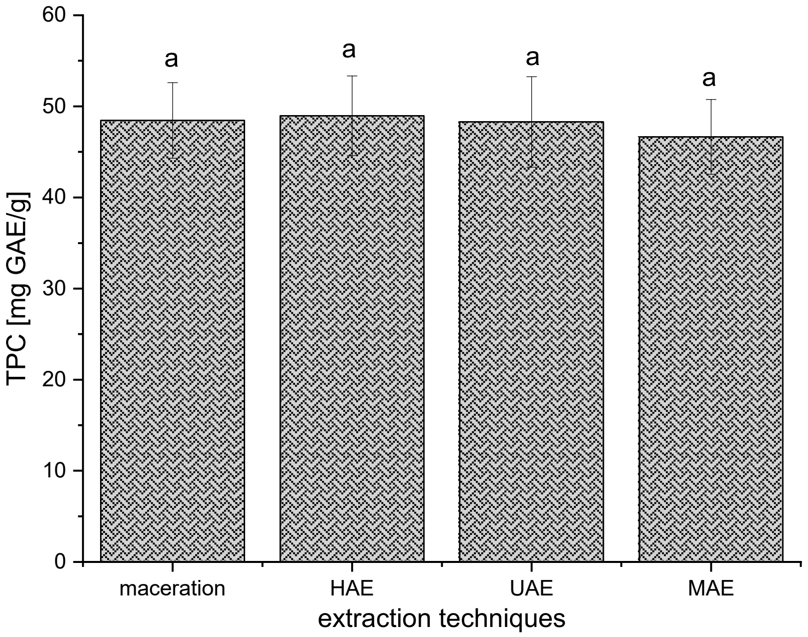

Figure S1. The influence of extraction procedures on total polyphenol content *Vaccinium myrtillus* leaf waste extracts from maceration, and heat, ultrasound, and microwave extractions (HAE, UAE, and MAE, respectively); the same letter represents the absence of significant differences based on one-way analysis of variance and Duncan's *post hoc* test at $p<0.05$; TPC, total polyphenol content; GAE, gallic acid equivalent.

Table S1. The optimization of maceration and heat, ultrasound, and microwave extractions (HAE, UAE, and MAE, respectively) of *Vaccinium myrtillus* by-product from leaves employing as a statistical tool a 2^3 experimental design.

	**Effect**	**Std. Err.**	**Effect Estimates**	**Coeff.**	**Std. Err. Coeff.**	***p***
**Maceration**						
TPC* (mg GAE/g)						
Constant				46.947	0.117	0.000
Main factors						
Solid-to-solvent ratio (g/mL) (1)	16.235	0.235	69.032	8.117	0.117	0.000
Solvent type (2)	0.610	0.235	2.594	0.305	0.117	0.019
Time (min) (3)	1.078	0.235	4.585	0.539	0.117	0.000
Interaction of two factors						
1 by 2	0.770	0.235	3.274	0.385	0.117	0.004
1 by 3	-0.028	0.235	-0.120	-0.014	0.117	0.905
2 by 3	-0.517	0.235	-2.197	-0.258	0.117	0.042
**HAE**						
TPC (mg GAE/g)						
Constant				46.881	0.077	0.000
Main factors						
Solid-to-solvent ratio (g/mL) (1)	17.736	0.153	115.725	8.868	0.077	0.000

Solvent type (2)	0.321	0.153	2.093	0.160	0.077	0.051
Time (min) (3)	0.067	0.153	0.440	0.034	0.077	0.665
Interaction of two factors						
1 by 2	0.214	0.153	1.397	0.107	0.077	0.180
1 by 3	-0.039	0.153	-0.256	-0.019	0.077	0.801
2 by 3	0.082	0.153	0.538	0.041	0.077	0.597
**UAE**						
TPC (mg GAE/g)						
Constant				46.449	0.114	0.000
Main factors						
Solid-to-solvent ratio (g/mL) (1)	17.320	0.229	75.647	8.660	0.114	0.000
Solvent type (2)	0.535	0.229	2.337	0.267	0.114	0.032
Time (min) (3)	1.807	0.229	7.891	0.903	0.114	0.000
Interaction of two factors						
1 by 2	0.180	0.229	0.786	0.090	0.114	0.443
1 by 3	0.528	0.229	2.307	0.264	0.114	0.034
2 by 3	0.193	0.229	0.844	0.097	0.114	0.410
**MAE**						
TPC (mg GAE/g)						
Constant				46.590	0.151	0.000
Main factors						
Solid-to-solvent ratio (g/mL) (1)	14.844	0.303	49.024	7.422	0.151	0.000
Solvent type (2)	-2.619	0.303	-8.650	−1.310	0.151	0.000
Time (min) (3)	10.237	0.303	33.810	5.119	0.151	0.000
Interaction of two factors						
1 by 2	0.616	0.303	2.034	0.308	0.151	0.058
1 by 3	2.736	0.303	9.035	1.368	0.151	0.000
2 by 3	−1.011	0.303	-3.338	-0.505	0.151	0.004

*TPC, total polyphenol content; GAE, gallic acid equivalent; the difference was statistically significant at $p<0.05$.

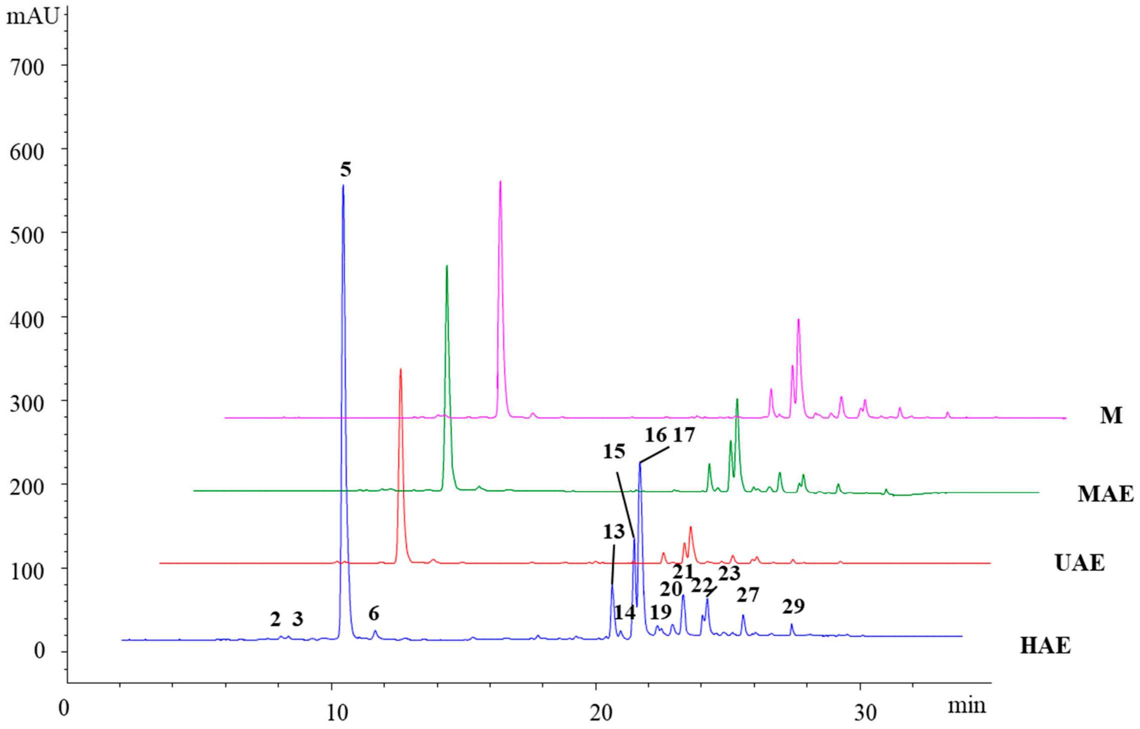

Figure S2. Chromatograms of four *Vaccinium myrtillus* extracts obtained from leaf dust by maceration, and heat, ultrasound, and microwave extractions (M, HAE, UAE, and MAE, respectively); numbers of compounds are given in Table 4.

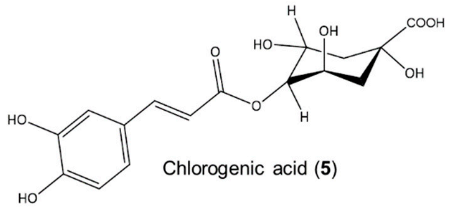

Chlorogenic acid (**5**)

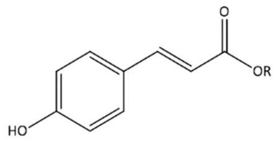

Compound	R
**19**	Malonyldihexose
**24, 26-28**	Malonylhexose
**25**	Diacetylhexose

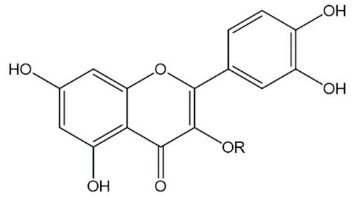

Compound	R
**13**	Rutinose
**15**	Galactose
**16**	Glucose
**17**	Glucuronic acid
**21**	Acetylhexose
**23**	Rhamnose
**29**	HMG-deoxyhexose

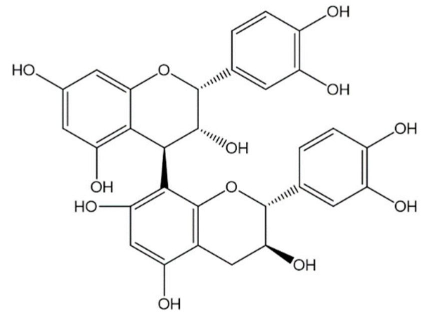

Procyanidin B2 (**7**)

Figure S3. Structures of the components identified in four *Vaccinium myrtillus* extracts from leaf dust using the LC-MS method; numbers of compounds are presented in Table 4.

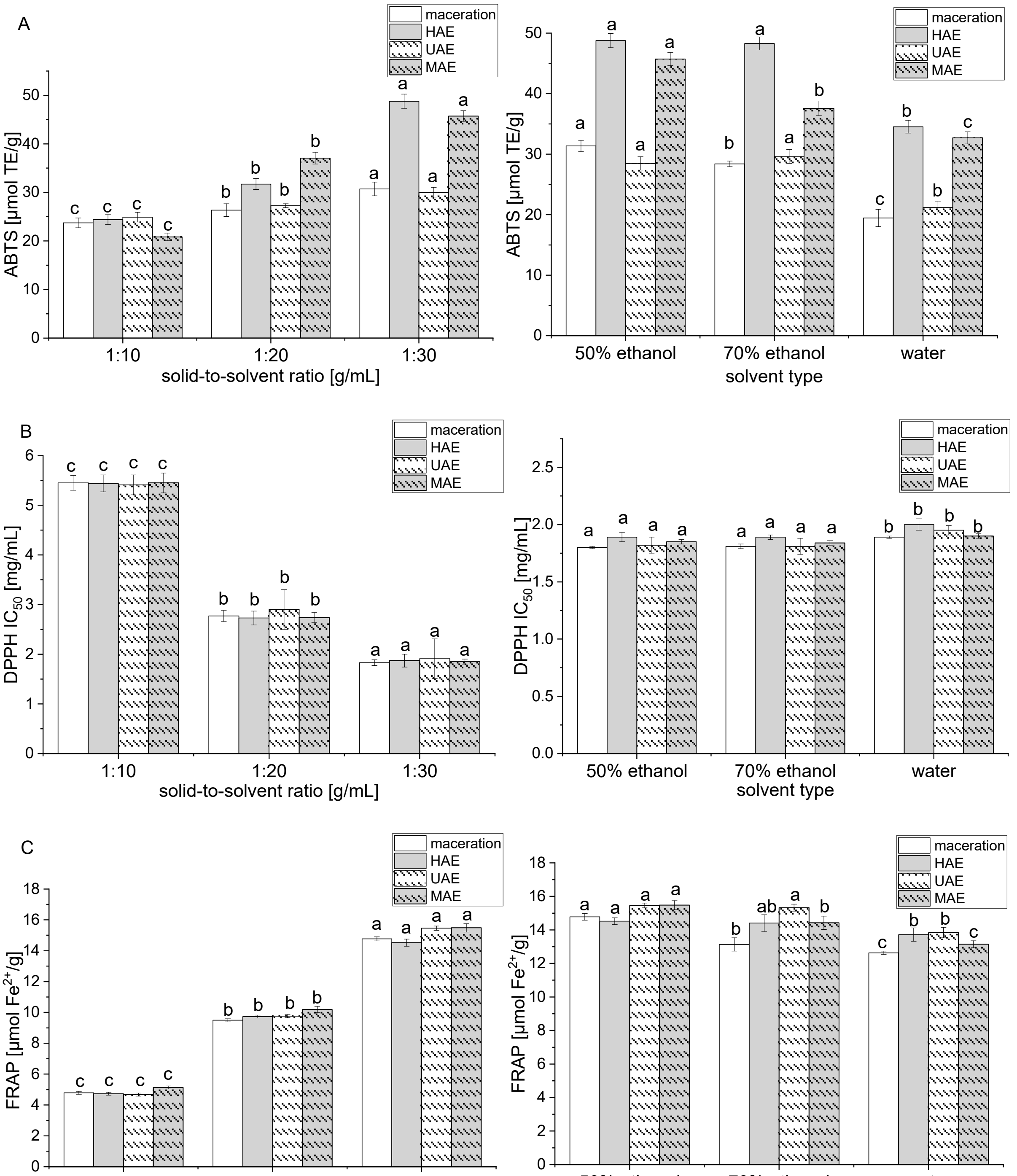

A
B
C
maceration
HAE
UAE
MAE
ABTS [µmol TE/g]
DPPH IC50 [mg/mL]
FRAP [µmol Fe2+/g]
1:10
1:20
1:30
solid-to-solvent ratio [g/mL]
50% ethanol
70% ethanol
water
solvent type

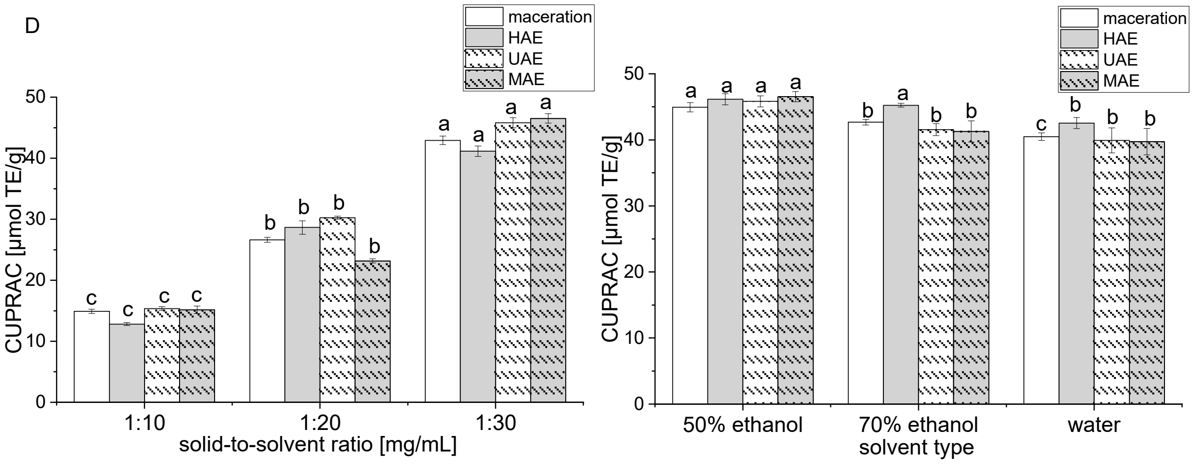

Figure S4. The effect of solid-to-solvent ratio and solvent type on (A) ABTS and (B) DPPH neutralization, (C) ferric ion-reducing potential, *i.e.*, FRAP, and (D) cupric ion-reducing activity, *i.e.*, CUPRAC of *Vaccinium myrtillus* extracts from waste of the leaves prepared using maceration, and heat, ultrasound, and microwave extractions (HAE, UAE, and MAE, respectively); different letters represent statistically significant difference between the parallels from the same extraction procedures based on one-way analysis of variance and Duncan's *post hoc* test (n=3; $p<0.05$); TE, Trolox equivalent.

Table S2. The effect of exposure period on ABTS and DPPH radical scavenging properties, ferric ion-reducing potential, *i.e.*, FRAP, and cupric ion-reducing activity, *i.e.*, CUPRAC of *Vaccinium myrtillus* extracts from maceration, and heat, ultrasound, and microwave extractions (HAE, UAE, and MAE, respectively).

Extraction techniques	Period (min)	antioxidant capacity			
		**ABTS (μmol TE/g)**	**DPPH IC_{50} (mg/mL)**	**FRAP (μmol Fe^{2+}/g)**	**CUPRAC (μmol TE/g)**
**Maceration**	30	30.80±1.10[a*]	1.90±0.16[a]	15.48±0.13[a]	42.15±1.31[b]
	45	31.36±1.18[a]	1.80±0.13[a]	15.33±0.41[a]	42.93±0.70[b]
	60	30.96±1.15[a]	1.81±0.11[a]	15.36±0.25[a]	45.65±0.43[a]
**HAE**	15	49.17±1.00[a]	1.82±0.10[a]	13.75±0.45[b]	40.50±0.51[a]
	30	49.81±1.13[a]	1.84±0.15[a]	14.98±0.47[a]	41.15±0.85[a]
	45	48.01±1.02[a]	1.86±0.09[a]	13.60±0.10[b]	40.47±1.08[a]
**UAE**	5	19.13±1.01[b]	1.84±0.11[a]	14.32±0.23[b]	34.58±0.57[c]
	15	29.96±1.11[a]	1.80±0.07[a]	15.14±0.45[a]	41.27±0.32[a]
	30	29.12±1.29[a]	1.81±0.14[a]	14.56±0.23[b]	39.82±0.48[b]
**MAE**	1	29.04±1.00[c]	1.89±0.03[a]	14.87±0.46[a]	43.35±1.20[b]
	2	45.72±1.08[a]	1.85±0.02[a]	15.48±0.11[a]	46.53±0.78[a]
	3	39.63±1.25[b]	1.85±0.04[a]	15.42±0.42[a]	40.20±0.85[c]

[*]Values with the same letter in each column showed no statistically significant difference ($p>0.05$; n=3; analysis of variance, Duncan's *post hoc* test); TE, Trolox equivalents; IC_{50}, the concentration of extract required to neutralize 50% of DPPH radicals.

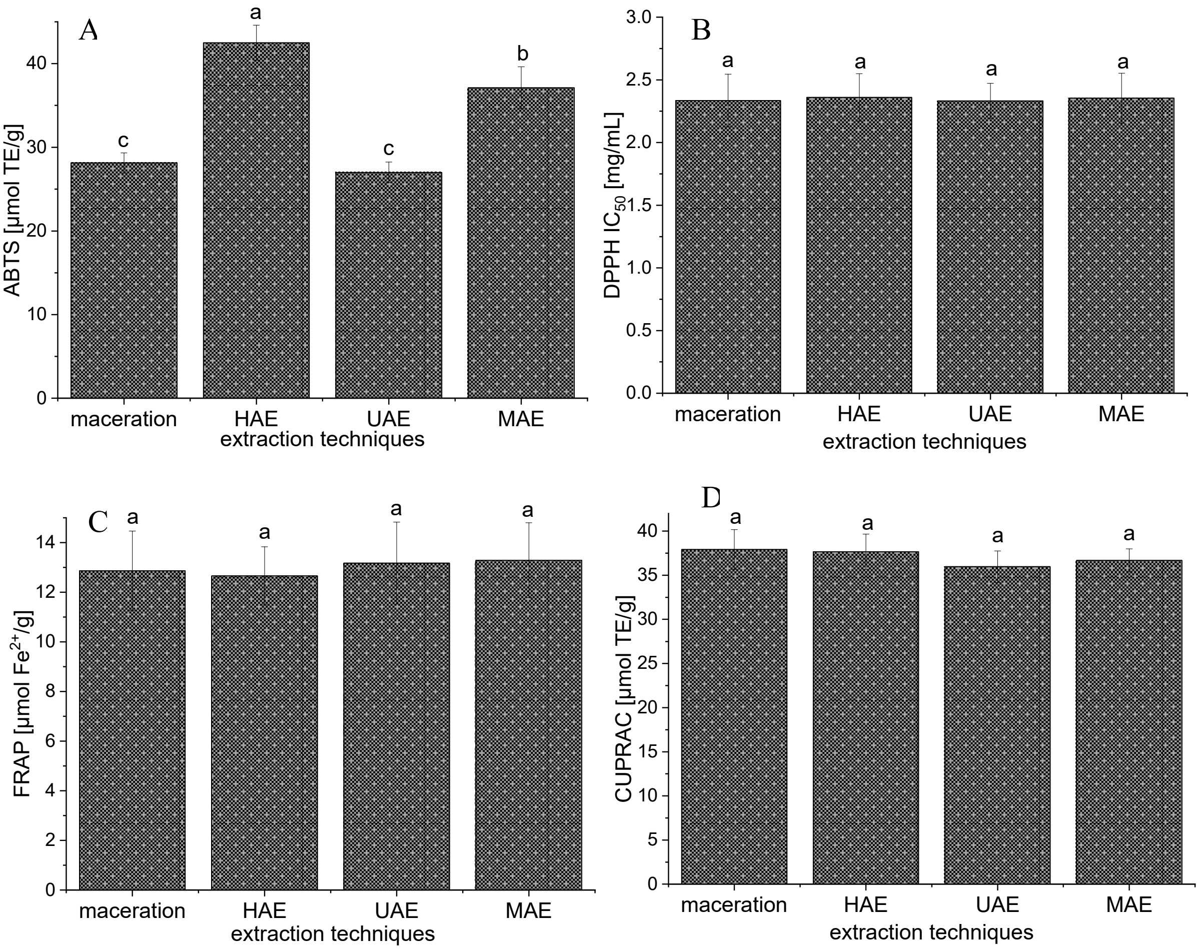

Figure S5. The impact of extraction methods on (A) ABTS and (B) DPPH neutralization, (C) ferric ion-reducing antioxidant power (FRAP), and (D) cupric ion-reducing activity (CUPRAC) of all obtained *Vaccinium myrtillus* extracts from maceration, and heat, ultrasound, and microwave extractions (HAE, UAE, and MAE, respectively); various letters mean different population groups based on one-way analysis of variance and Duncan's *post hoc* test (n=3; $p<0.05$); TE, Trolox equivalents; IC_{50}, the concentration of extract required to neutralize 50% of DPPH radicals.

Table S3. The antibacterial and antifungal potential of four *Vaccinium myrtillus* extracts obtained using leaf by-product and maceration, and heat, ultrasound, and microwave extractions (HAE, UAE, and MAE, respectively) investigated in the disk diffusion assay.

Microorganism	maceration	HAE	UAE	MAE	antibiotics/fluconazole
	zone of inhibition (cm)				
Staphylococcus aureus	1.9*	1.8	1.9	1.8	S
Enterococcus faecalis	1.0	1.5	1.3	1.0	S
Escherichia coli	R	R	R	R	R/I/S
Pseudomonas aeruginosa	R	R	R	R	S/I
Klebsiella spp.	R	R	R	R	S/I
Proteus spp.	R	R	R	R	S/I
Candida albicans	R	R	R	R	S

*The analysis was performed in triplicate and the lowest obtained value of the inhibition zone was taken as the result ("stricter criteria").

Table S4. The data of *Vaccinium myrtillus* leaf dust extracts' extraction yield (EY), electrolytic conductivity (*G*), density (ρ), surface tension (γ), and viscosity (η).

extraction	EY (%)	*G* (mS/g)	ρ (g/mL)	γ (mN/m)	η (mPa·s)
maceration	$1.58 \pm 0.14^{ab*}$	0.28 ± 0.02^{b}	0.961 ± 0.008^{a}	27.4 ± 1.3^{ab}	3.12 ± 0.13^{a}
HAE	1.14 ± 0.20^{c}	0.20 ± 0.02^{c}	0.919 ± 0.006^{c}	26.2 ± 0.1^{b}	2.99 ± 0.13^{a}
UAE	1.31 ± 0.29^{bc}	0.18 ± 0.02^{c}	0.968 ± 0.019^{a}	28.5 ± 0.4^{a}	3.19 ± 0.14^{a}
MAE	1.94 ± 0.27^{a}	0.35 ± 0.03^{a}	0.930 ± 0.009^{b}	26.1 ± 1.0^{b}	2.96 ± 0.20^{a}

*Values with the same letter in each column showed no statistically significant difference ($p>0.05$; n=3; analysis of variance, Duncan's *post hoc* test); heat-, ultrasound- and microwave-assisted extractions (HAE, UAE, and MAE, respectively).
